# Redox-State-Dependent Structural Changes within a Prokaryotic 6–4 Photolyase

**DOI:** 10.1021/jacs.4c18116

**Published:** 2025-04-29

**Authors:** Po-Hsun Wang, Yuhei Hosokawa, Jessica C Soares, Hans-Joachim Emmerich, Valeri Fuchs, Nicolas Caramello, Sylvain Engilberge, Andrea Bologna, Christian Joshua Rosner, Mai Nakamura, Mohamed Watad, Fangjia Luo, Shigeki Owada, Takehiko Tosha, Jungmin Kang, Kensuke Tono, Yoshitaka Bessho, Eriko Nango, Antonio J. Pierik, Antoine Royant, Ming-Daw Tsai, Junpei Yamamoto, Manuel Maestre-Reyna, Lars-Oliver Essen

**Affiliations:** † Department of Chemistry, Philipps University Marburg, Hans-Meerwein Strasse 4, Marburg 35032, Germany; ‡ Institute of Biological Chemistry, Academia Sinica, 128 Academia Rd. Sec. 2, Nankang, Taipei 115, Taiwan; § Department of Chemistry, National Taiwan University, 1, Roosevelt Rd. Sec. 4, Taipei 106, Taiwan; ∥ Division of Chemistry, Graduate School of Engineering Science, Osaka University, 1-3 Machikaneyama, Toyonaka, Osaka 560-8531, Japan; ⊥ Biochemistry, Faculty of Chemistry, University of Kaiserslautern, Kaiserslautern D-67663, Germany; # European Synchrotron Radiation Facility, 38043 Grenoble, France; ∇ Hamburg Centre for Ultrafast Imaging, University of Hamburg, 22761 Hamburg, Germany; ○ Univ. Grenoble Alpes, CNRS, CEA Institut de Biologie Structurale, 38044 Grenoble, France; ◆ Department of Science and Technology, University of Sannio, Via Francesco de Sanctis, snc, 82100 Benevento, Italy; ¶ Japan Synchrotron Radiation Research Institute, 1-1-1 Kouto, Sayo-cho, Sayo-gun, Hyogo 679-5198, Japan; †† RIKEN SPring-8 Center, 1-1-1 Kouto, Sayo-cho, Sayo-gun, Hyogo 679-5148, Japan; ‡‡ Graduate School of Agricultural and Life Sciences, University of Tokyo, 1-1-1 Yayoi, Bunkyo, Tokyo 113-8657, Japan; §§ Institute of Multidisciplinary Research for Advanced Materials, Tohoku University, 2-1-43 1 Katahira, Aoba-ku, Sendai 980-8577, Japan

## Abstract

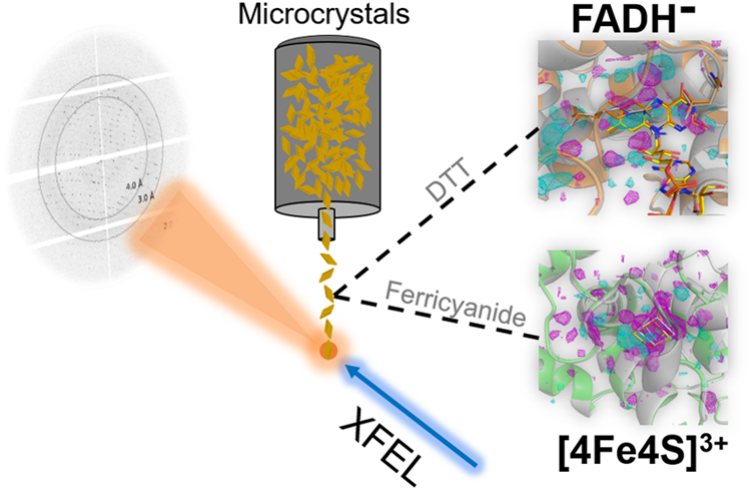

Photolyases repair UV damage to DNA by using absorbed blue light. Within the photolyase/cryptochrome superfamily (PCSf), a major subgroup consists of prokaryotic (6–4) photolyases. These enzymes rely on flavin adenine dinucleotide (FAD) as a catalytic cofactor, besides an ancillary antenna chromophore, and a [4Fe-4S] cluster with yet unknown function. For the prokaryotic 6–4 photolyase of *Caulobacter crescentus*, we investigated structural changes associated with its different redox states by damage-free crystallography using X-ray free-electron lasers. EPR and optical spectroscopy confirmed redox-dependent structural transitions, including the formation of an oxidized [4Fe-4S]^3+^ cluster with the dynamic cleavage of a single iron–sulfur bond. Photoreduction to the catalytic FADH^–^ state alters the flavin binding site at the proximal aromatic pair Y390/F394 that is part of the electron transport pathway. Upon oxidation, the observable structural transitions of the protein matrix around the [4Fe-4S] cluster may affect DNA binding and are consistent with the much-debated role of the iron–sulfur cluster in DNA-binding proteins for quenching electron holes.

## Introduction

Light plays a crucial role as an environmental cue in regulating cellular responses. Various classes of photoreceptors have been identified, absorbing light energy across different spectral regions to modulate organismic functions. Among these, photolyases (PLs) and cryptochromes (CRYs) are known to absorb near-UV to blue light within the wavelength range of 300 to 500 nm.^1^ PLs are photoactive enzymes that facilitate the repair of DNA damage caused by exposure to the sun’s ultraviolet (UV) radiation. Specifically, PLs are responsible for repairing two types of UV-induced DNA lesions: cyclobutane-pyrimidine dimers (CPDs) and pyrimidine-(6–4)-pyrimidone photoproducts (6–4PPs).^2−4^ While most CRYs primarily function as signaling proteins, some CRYs are bifunctional by acting also as PL.^5^ CRY-dependent responses include entrainment of the circadian clock, developmental adaptation, and even magnetoreception.^6,7^ PPLs and CRYs share an evolutionary relationship and possess a common architecture that is characterized by the presence of a U-shaped FAD chromophore in the C-terminal α-helical domain. Both CRYs and PLs use photoreduction and intramolecular light-driven electron transfer (ET) reaction to produce reduced FAD states—either for signaling (FAD^•–^, FADH^•^) in CRY or for achieving catalytic competence (FADH^–^) in PL.

The ET pathway for FAD photoreduction consists of an aromatic triad found to be highly conserved within PCSf subfamilies, e.g., among class I CPD, DASH and eukaryotic (6–4) PLs, but different for other subfamilies, e.g., when comparing class I with class II PL.^2,8,9^ During photoreduction, the aromatic triad facilitates electron transfer from its final, surface-exposed aromatic residue to the inactive oxidized FAD, resulting in the ultrafast formation of a transient FAD^•–^/Ar^•^ radical pair. FAD^•–^ may then become protonated to form the long-lived FADH^•^ state.^10,11^ In PLs, a second round of ET results in a fully reduced state (FADH^–^). This state is crucial in catalyzing the repair of cyclobutane pyrimidine dimers (CPD) or (6–4) photoproducts in nucleic acids. Here, the reaction is initiated by electron injection from the light-excited FADH^–*^ to the DNA damage.^3,12^

Crystal structures of members from different PCSf subfamilies exhibit striking similarities in their overall structural properties. The C-terminal Photolyase homology region (PHR) domain houses the FAD chromophore, while the N-terminal region comprises a Rossman-like α/β domain. This N-terminal region often serves as a binding site for a second ancillary chromophore, acting as an antenna to enhance absorption in the visible range.^13,14^ Several antenna chromophores absorbing within the spectral region of 380 to 420 nm are known, including 8-hydroxydeazaflavin (8-HDF), FAD, flavin mononucleotide (FMN), 5,10-methenyltetrahydrofolate (MTHF), and 6,7-dimethyl-8-ribityllumazine (DLZ).^5,13−16^

In the PCSf, the subfamily of prokaryotic (6–4) photolyase contains an additional cofactor, [4Fe-4S], and was initially coined as Fe-S containing bacterial cryptochromes and photolyases (FeS-BCP).^16,17^ However, [4Fe-4S] clusters may not be required for DNA repair by all prokaryotic (6–4) PL, as several orthologs miss cysteines coordinating this metal cluster.^18^ Iron–sulfur clusters are involved in ET processes during photosynthesis, respiration, and nitrogen fixation.^19,20^ Additionally, several DNA repair enzymes, including DNA helicases,^21^ MutY,^22^ and endonuclease III,^23^ also harbor iron–sulfur clusters. MutY and endonuclease III likely utilize their [4Fe-4S] cluster to effectively identify and locate DNA lesions.^24^ Moreover, many iron–sulfur clusters are unstable in the presence of molecular oxygen. Under aerobic conditions, oxygen-sensitive [4Fe-4S] clusters can degrade into [3Fe-4S] clusters and may further decompose into [2Fe-2S] clusters.^25,26^ For example, [4Fe-4S]^2+^ clusters in dehydratases are highly susceptible to oxidation, leading to their damage when exposed to oxygen.^27^ However, the function of [4Fe-4S] cluster in prokaryotic (6–4) PL remains unclear, although it has been discussed to act as a cache for electrons.^28^

X-ray crystallography, as a technique for obtaining high-resolution structures of metalloproteins, often suffers from radiation damage at metal centers, affecting their redox state. The advent of X-ray free-electron lasers (XFEL) solved this limitation, as XFELs deliver intense and ultrashort X-ray pulses of less than 10 fs, allowing the damage-free collection of diffraction data from microcrystals before their destruction.^29,30^ In our study, the first damage-free crystal structure of a prokaryotic (6–4) photolyase, *Cc*(6–4)PL, was obtained by serial femtosecond crystallography (SFX). We further established the fully reduced state of *Cc*(6–4)PL to investigate structural changes upon the photoreduction to the catalytically active state. Moreover, due to the potential sensitivity of the built-in [4Fe-4S]^2+^ cluster for oxidation, we generated the oxidized [4Fe-4S]^3+^ species to explore the structural consequences of oxidation. This work lays the groundwork for future time-resolved experiments on *Cc*(6–4)PL during light-driven cofactor reduction.

## Materials and Methods

### *Cc*(6–4)PL Overproduction and Purification

*Escherichia coli* BL21(DE3) was transformed with a pET28-based construct coding for the *Cc*(6–4)PL (Uniprot entry Q9AAF5). *Cc*(6–4)PL was overproduced by autoinduction in Terrific Broth medium at 25 °C for 27 h. Centrifuged cell pellets were then resuspended in buffer A (50 mM NaH_2_PO_4_, 100 mM NaCl, 20% glycerol, pH 8), followed by the addition of a spatula tip of lyophilized DNase I, lysozyme, and containing 0.2 mM PMSF. Cell disruption was performed with a high-pressure homogenizer (EmulsiFlex-C3, Avestin) at 4 °C, followed by centrifugation to remove cell debris. As the first purification step for *Cc*(6–4)PL, the supernatant was loaded onto a self-packed 10 mL nickel-NTA (Roche) column pre-equilibrated with buffer A at 4 °C. The nickel-NTA matrix was sequentially washed by 5 column volumes (CV) of buffer A and 5 CVs washing buffer (buffer A supplemented with 25 mM imidazole). *Cc*(6–4)PL was eluted with buffer A supplemented with 250 mM imidazole. Protein-containing fractions were then pooled and applied to a heparin affinity column (HiTrap 5 mL Heparin HP, GE Healthcare). The column was washed with 5 CVs buffer A, before *Cc*(6–4)PL was eluted with buffer B (50 mM NaH_2_PO_4_, 250 mM NaCl, 20% glycerol, pH 8). Thereafter, size exclusion chromatography was performed at 4 °C as the final purification step. A HiLoad 16/600 Superdex 200 (GE Healthcare) was used and equilibrated in gel filtration buffer (20 mM HEPES, 200 mM NaCl, and 5% glycerol, pH 7.8). *Cc*(6–4)PL purification was monitored by SDS-PAGE (Figure S1A,B). In general, purified *Cc*(6–4)PL was produced with yields of ∼50 mg/L expression culture.

### Production of *Cc*(6–4)PL Microcrystals for SFX

Initial crystallization screening of *Cc*(6–4)PL at a protein concentration of 16 mg/mL (Amicon, 30K) was carried out in a 96-well format with several commercially available crystallization screens. The found conditions were optimized by the sitting drop technique at 20 °C. Macroscopic *Cc*(6–4)PL crystals, i.e., with dimensions of up to 120 μm, were obtained after 4 days in JCSG Core II (1.0 M LiCl; 0.1 M MES, pH = 6.0; 20% (w/v) PEG 6000) (Figure S1C). However, to perform SFX experiments, we rescreened conditions for obtaining large quantities of microcrystals (∼10 μm). For that, *Cc*(6–4)PL was concentrated to 3 mg/mL and sterile-filtered under safety light to prevent any light-induced reaction. *Cc*(6–4)PL microcrystals were obtained in 0.1 M Tris, pH 8.5, 20% PEG 3350, 0.2 M MgCl_2_ at 23 °C by using the microseeding method (Hampton Research). Briefly, a 1 mL crystal seeding tube was prepared in advance. First, 10 μL of crystals was pipetted from a stock crystallization batch and added to 990 μL of the crystallization mixture comprising 1:1 protein solution: crystallization buffer. The crystals were then crushed for 5 min by vortex and pausing every 30 s. Large-scale microcrystallization in a Falcon tube was achieved by mixing a 1:1 ratio of protein and crystallization buffer with 1% (v/v) seed stock from the seeding tube. Finally, the tube was wrapped with aluminum foil and incubated for at least 8 h before crystals were formed (Figure S1D).

### Synchrotron-Based Data Collection and Structure Solution

X-ray data were collected from single crystals at 100 K at beamline X06SA (PXI) at the Swiss Light Source (SLS, Villigen, Switzerland) initially. Furthermore, data from microscopic crystals were collected at 293 K at the beamline (TPS05A) at National Synchrotron Radiation Research Center (NSRRC, Hsinchu, Taiwan). The diffraction data sets were processed by XDS.^31^*Cc*(6–4)PL structures were solved via molecular replacement by Phaser,^32^ using *Vc*(6–4)PL (PDB entry: 8A1H)^33^ as a homology model of *Cc*(6–4)PL. Further refinements were accomplished by a combination of phenix.refine from Phenix,^34^ REFMAC5^35^ from the CCP4 software package,^32^ and Coot.^35^ Likewise, a data set was collected for the K48A mutant at beamline ID23–2 at the European Synchrotron Radiation Facility (ESRF, Grenoble, France). Anisotropic B-factor refinement was confined in all cases to the heavy atoms of the [4Fe-4S] cluster; otherwise, individual, isotropic B-factor/TLS refinement was done for the protein chain. Data collection, processing, and refinement details can be found in Tables S1 and S5.

### FADH^–^ and [4Fe-4S]^3+^ States of *Cc*(6–4)PL Crystals

To avoid reoxidation upon photoreduction, all buffers were degassed and incubated in an anaerobic chamber (COY) for 24 h. An open container was used to put the crystal aliquot and 77 mg of powdered DTT into the anaerobic chamber airlock. Afterward, the aliquots were subjected in the airlock to seven cycles of evacuation and nitrogen flushing, followed by storage at a reduced pressure of 68 mbar for 1 h. After evacuation and flushing the airlock twice with working gas mix (96% N_2_, 4% H_2_), both samples were placed into the anaerobic chamber and the crystals on a Lab-Armor bead-filled dry bath at 23 °C. To obtain fully reduced *Cc*(6–4)PL, 77 mg of powdered DTT was mixed with 1 mL of degassed gel filtration buffer to make a new 0.5 M DTT solution under anaerobic conditions. Mixing 2.7 mL of crystals with 300 μL of DTT solution generated a final concentration of 50 mM DTT. Finally, a white light source and blue LED were positioned to cover the whole tube, which was illuminated for 30 min to generate the fully reduced state.

To produce the oxidized [4Fe-4S]^3+^ state, 1.2 μL of 0.5 M potassium ferricyanide (K_3_FeCN_6_) was added to 300 μL of the *Cc*(6–4)PL crystal suspension.^16^ Thereafter, the mixture was incubated at room temperature for 5 min to produce the oxidatively damaged [4Fe-4S]^3+^ state of *Cc*(6–4)PL (superoxidized state).

### *Cc*(6–4)PL Crystal Embedding and Injector Building

The *Cc*(6–4)PL crystal suspension was concentrated by centrifugation at 8000 rpm for 5 min, resulting in the fully compacted crystal slurry. The supernatant was removed as much as possible, and crystal slurry was embedded in a 1:9 ratio with a hydrophobic grease matrix as previously described.^36^ Next, the embedded material was transferred to a φ4 mm cartridge using a flat spatula before the Teflon cylinder was inserted into the top of the cartridge. The injector was then assembled with the cartridge, the O-ring, and a 75 μm nozzle and then wrapped in an opaque container to prevent light exposure during transport to the hutch.

### SFX Data Collection and Data Processing at SACLA

All experiments took place at the SACLA BL2 beamline inside the DAPHNIS system.^37^ Here, the primary parts are the helium chamber, injectors, and the MPCCD detector with a 50 mm distance to the sample.^38^ Helium gas contents of >98% within the DAPHNIS system prevented *in situ* oxidation; a water circulation system maintaining 20 °C avoided sample heating during extrusion. In *Cc*(6–4)PL experiments, a 75 μm nozzle was used with a 1 μL/min flow rate for SFX. Images were captured utilizing 10 keV X-ray pulses with a duration of <10 fs and 1.5 μm beam diameter. In order to compare differences between dark-adapted, i.e., the oxidized state, and other redox states, at least 50,000 indexed images were collected to obtain high-quality data sets. The Cheetah pipeline of SACLA^39^ evaluated images in real time for diffraction patterns (hits). After data collection, the offline pipeline initiated automated spot finding and indexing, converted hit images into HDF5 files, and sent the data to CrystFEL.^40^ CrystFEL generated preliminary data sets to evaluate data quality, light contamination, detector geometry optimization, etc. *Cc*(6–4)PL data sets were combined by *process_hkl* and afterward converted into the MTZ file format, which can be handled within the CCP4 suite.

### Refinement of Structures from SFX Data Sets

SFX data sets were solved by molecular substitution using the synchrotron structure of *Cc*(6–4)PL (FAD_ox,sync2_ state) as start model. After refinement of the FAD_ox_ state structure, initial rigid-body and restrained refinement of other SFX data sets utilized this structure thus resulting in basal models for all redox-state data sets. To determine the occupancies of the fully reduced and oxidatively damaged structures, experimental structure factors (Fo) were deconvoluted using an established extrapolation protocol:^36^ Using the dark-adapted data set for the FAD_ox_ state, the CCP4 suite’s *SFALL* program^32^ generated a set of calculated structure factors (Fc_dark_) and phases (PHIc_dark_) for the dark structure. Then, the CCP4 suite’s SCALEIT program scaled dark observed structure factors (Fo_dark_) and the redox-state-dependent structure factors X (Fo_*x*_) against Fc_dark_. After calculating Bayesian and occupancy-weighted normalized difference structure factors (dFo_WN_), Fc_dark_ was used to generate extrapolated structure factors (*F*_ext_). To estimate the occupancy parameter (*N*), extrapolation was performed at several *N* values until an inflection point was detected in the integrated residual negative density of an area of interest (Figure S2), which was the FAD cofactor for the fully reduced state and the [4Fe-4S]-cluster for the superoxidized state, respectively. Subsequently, basal models were further refined as before against the occupancy-adjusted extrapolated data sets. To determine the precise FAD geometry, difference map real space correlation coefficient (dFoCC) refinement was performed, as previously described.^41^ The final statistics of data collection, processing, and refinement are summarized in Table S1.

### Generating *Cc*(6–4)PL Difference Electron Density Maps

The isomorphous difference electron density maps were directly calculated from experimental data via the phenix.fobs_minus_fobs_map tool.^42^ Resolution limits were set to the lower of the two data sets for high resolution and 10 Å for low resolution.

### Electron Paramagnetic Resonance (EPR) Spectroscopy

EPR samples of *Cc*(6–4)PL (80 μM) under reducing conditions were obtained by anaerobically incubating 300 μL of protein with either 50 mM DTT or 2 mM sodium dithionite and blue light illumination for 30 min. The protein in the superoxidized state was prepared by aerobically incubating 300 μL of protein with 2 mM K_3_FeCN_6_ for 3 min. The FADH^•^ state sample was prepared by blue light illumination for 30 min under aerobic conditions. The “as isolated” sample, i.e., the FAD_ox_ state, consisted of 300 μL of protein under aerobic conditions without any additional treatment. After sample preparation in Eppendorf tubes, each sample was loaded into a quartz EPR tube and shock-frozen in liquid nitrogen. EPR spectra were recorded with an Elexsys E580 Bruker X-band spectrometer equipped with a 4122HQE resonator. Data collection and conversion to ASCII format were performed using the manufacturer’s XEPR program. Data processing was done using Microsoft Excel.

### *In Crystallo* UV–Vis Absorption Spectroscopy

*In crystallo* UV–vis absorption spectroscopy was accomplished at the *ic*OS Laboratory, ESRF, Grenoble, France.^43^*Cc*(6–4)PL microcrystal slurries were photoreduced into the FADH^•^ and FADH^–^ redox states like in the steady-state XFEL studies. Under a constant nitrogen gas flow, samples were immediately mounted on the goniometer of the main *ic*OS setup at 100 K. UV–vis data were obtained using the 50 μm focal point of the incoming objective, to which the probing white light lamp was connected via a 200 μm optical fiber. The detector optics were linked to the outgoing objective via a 400 μm diameter optical fiber. The duration of each acquisition was 200 ms; each spectrum was obtained by averaging 100 acquisitions. The contribution of Rayleigh scattering was subtracted from each spectrum, then each spectrum was scaled on the absorbance at 280 nm, using the *ic*OS-toolbox software developed at the ESRF (see https://github.com/ncara/icos).

## Results

### Determination of [4Fe-4S] Redox States in *Cc*(6–4)PL

**Figure 1:**
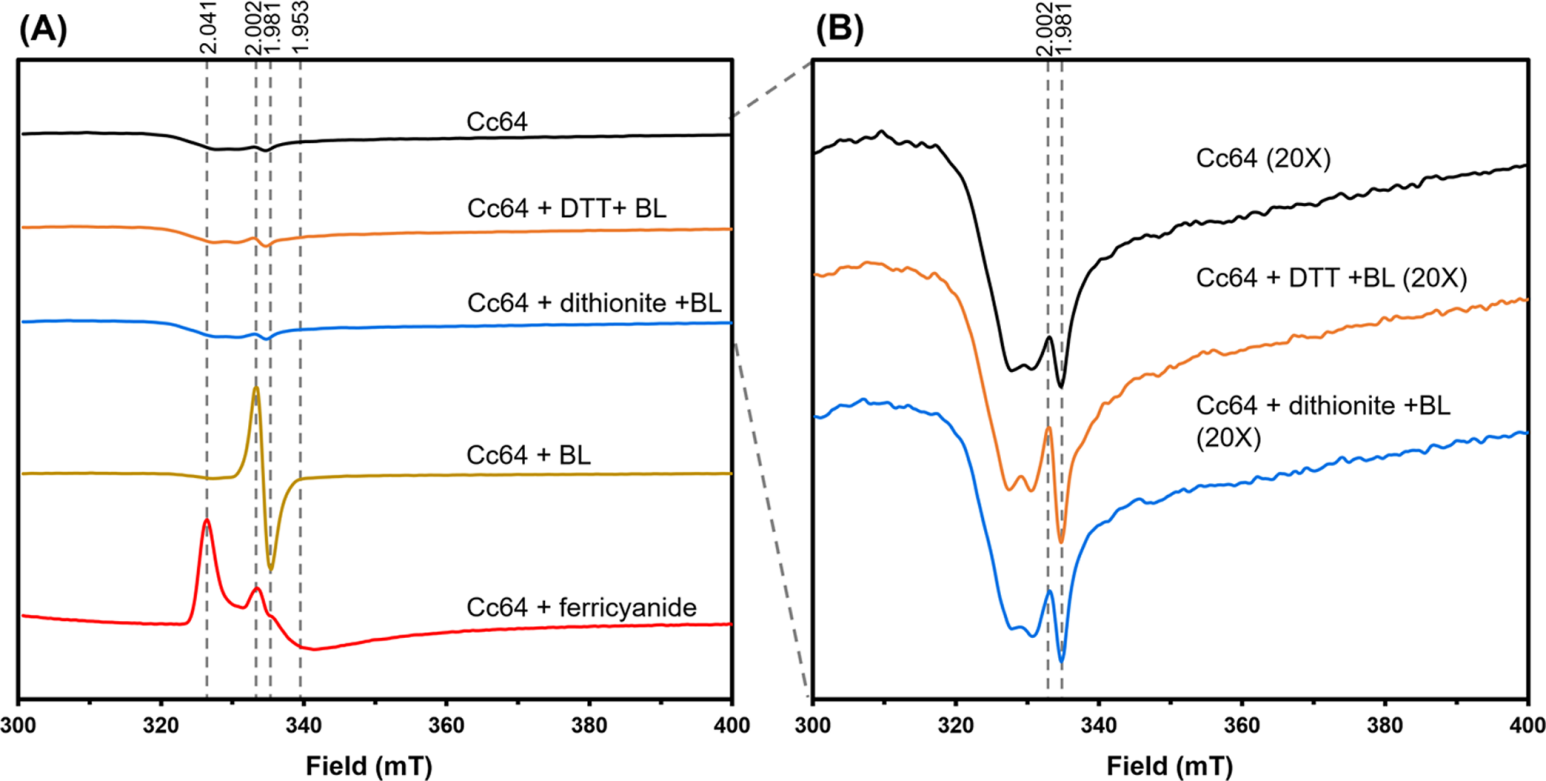
X-band EPR spectra of different redox states of *Cc*(6–4)PL at 10 K. (A) Coloring of the EPR signals with *Cc*(6–4)PL (black), with 50 mM DTT (orange), with 2 mM dithionite (blue), with 455 nm blue light (BL) for 30 min (gold), and 2 mM ferricyanide for 3 min (red). (B) Close view of EPR-silent samples. EPR conditions: microwave power, 0.2 mW; modulation amplitude, 1.0 mT; modulation frequency, 9.35 kHz. Treatment of *Cc*(6–4)PL with ferricyanide did not affect the EPR-silent oxidized state of the FAD chromophore, as also shown by UV/vis data (Figure S4A).

The activity profile of *Cc*(6–4)PL corresponds closely to that of other prokaryotic (6–4) PLs as shown by *in vitro* DNA repair assays (Figure S3). Accordingly, DNA repair highly depended on the presence of Mg^2+^ ions, which increased the repair activity by 9-fold. As putative Mg^2+^ ion binding sites within prokaryotic (6–4) PLs are highly conserved, two crucial aspartic acid residues, D178 and D253, within *Cc*(6–4)PL can be predicted to coordinate Mg^2+^ as a catalytic cofactor. Another hallmark of prokaryotic (6–4) PLs is the presence of a [4Fe-4S]^2+^ cluster in the oxidized FAD and reduced FADH^–^ states.^16,17^ To confirm the [4Fe-4S] redox states in the different redox states of *Cc*(6–4)PL, we employed EPR spectroscopy (Figure 1A). In its FAD_ox_ state, *Cc*(6–4)PL lacked any significant EPR signals by displaying only a very weak organic radical signal from minor contamination by the FADH^•^ state (Figure 1B). This indicates that *Cc*(6–4)PL contains solely [4Fe-4S]^2+^ when isolated, as the [4Fe-4S]^2+^ state is diamagnetic and the EPR is silent. Upon blue light illumination, a strong radical signal typical of the FADH^•^ state with a line width of 1.9 mT was detected.^44^ Accordingly, no EPR signals were observed when the samples were photoreduced to the fully reduced FADH^–^ state using 455 nm blue light for 30 min under anaerobic conditions and with the reducing agents DTT or dithionite (see Figure S4 for UV–vis spectroscopy). These results indicate that reductants affect only the redox state of FAD, but not that of the [4Fe-4S] cluster.

Interestingly, treatment with the oxidizing agent ferricyanide produced a strong EPR signal indicative of spin-coupled paramagnetic ions with *g*-values of 2.04, 2.002, and 1.953. Signals with *g*-values near 2.0 are characteristic of iron–sulfur proteins in oxidized states, such as [3Fe-4S]^1+^ or [4Fe-4S]^3+^ clusters. However, the observed EPR signals upon oxidation are distinct from those noted for [3Fe-4S]^1+^ clusters, e.g., in aconitase.^45^ Instead, the signals resemble more of [4Fe-4S]^3+^ clusters, with spectral properties being similar but not identical to high-potential iron–sulfur proteins (HiPIPs),^46^ as these also produce EPR signals in their oxidized state with g-factors around 2.05. Unlike HiPIPs, the oxidation toward the [4Fe-4S]^3+^ state proceeds in *Cc*(6–4)PL incomplete and may be subject to a dynamic equilibrium. Using HiPIP spectra from Ohno et al.^47^ (Figure S4B) and reported molar absorption coefficients of ∼16,000 M^–1^ cm^–1^ for the [4Fe-4S]^2+^ cluster, one can derive a Δε_470_ of ∼8400 M^–1^ cm^–1^ for the oxidation to the [4Fe-4S]^3+^ state.^48^ With an ε_470_ of ∼7500 M^–1^ cm^–1^ for FAD_ox_, one can estimate from our difference *Cc*(6–4)PL difference spectra (Figure S4A) that only 6% of the [4Fe-4S] cluster assumes the +3 state under these conditions.

### Damage-Free *Cc*(6–4)PL Structures in Various Redox States

**Figure 2:**
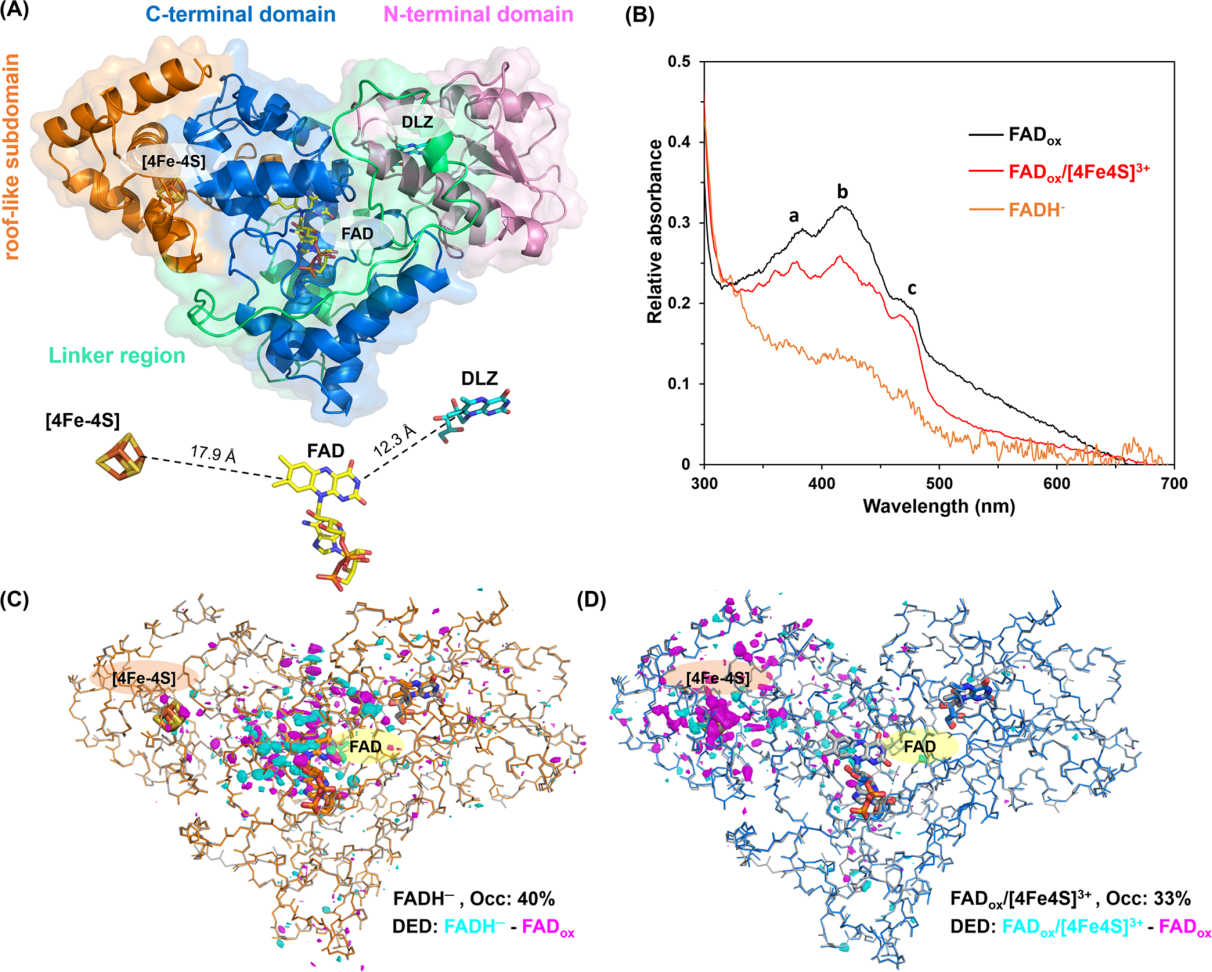
*Cc*(6–4)PL structures of its dark-adapted and oxidized state (FAD_ox_/[4Fe-4S]^2+^) and redox-state-dependent structural changes. (A) Overall protein fold of the dark-adapted and oxidized state (FAD_ox_) is shown with the N-terminal α/β domain in pink, the linker region in green, the C-terminal α-helical domain in blue, and roof-like subdomain in orange. The three cofactors are shown as stick model both within the protein moiety and separately; DLZ in cyan, FAD in yellow, [4Fe-4S] cluster in orange. The shown values are the shortest distances between the chromophores, measured from one edge to another in the right panel. (B) *In crystallo* spectra of FAD_ox_ (black line), FAD_ox_/[4Fe-4S]^3+^ (red line), and FADH^–^ (orange line) adopted by *Cc*(6–4)PL crystals. The spectra of FAD_ox_ and FAD_ox_/[4Fe-4S]^3+^ were taken in the dark. The spectrum of FADH^–^ was obtained after light illumination for 30 min with 50 mM DTT. The FAD characteristic absorption peaks within the blue region of the spectrum are labeled at certain wavelengths, namely *a* = 382 nm, *b* = 440 nm, and *c* = 475 nm. (C) To highlight the major differences between FAD_ox_ and FADH^–^ structures, the FAD_ox_ state backbone is shown as line model (gray) behind the FADH^–^ state model (orange), with cofactors shown as stick models and labeled [4Fe-4S] and [FAD]. A 3.5 σ contoured DED map comparing the two states is overlaid (cyan: positive peaks; magenta: negative peaks). Major structural changes in the FADH^–^ state occur around the catalytic FAD chromophore. (D) Structural differences between the FAD_ox_ state (gray) and its FAD_ox_/[4Fe-4S]^3+^ state (blue) shown as in (C). Here, structural changes are mostly around the [4Fe-4S] cluster.

The *Cc*(6–4)PL structure, solved by SFX at room temperature and in darkness, comprises an α/β-domain at the N-terminus and an all-α-helical domain at the PHR domain (Figures 2A and S5A). The N-terminal region (M1-T125) contains four parallel β-strands surrounded by α-helices and houses the DLZ antenna chromophore. The C-terminal region (P230–S508) consists of 16 α-helices and connects to the α-β domain via a long linker (R126-N229). FAD, its built-in cofactor, adopts a U-shaped conformation. Within the α-helical domain, a roof-like subdomain formed by 4 α-helices (P454–S508) harbors the [4Fe-4S] cluster as a third cofactor. The distance from the catalytic FAD to the cluster is 17.9 Å, while the DLZ antenna is closer to the FAD, with an edge-to-edge distance of 12.3 Å (Figure 2A).

To validate the different redox states spectroscopically *in crystallo*, *Cc*(6–4)PL crystals underwent the same treatment as those subsequently used in steady-state SFX experiments (Figure 2B). In the superoxidized state—when *Cc*(6–4)PL with its bound FAD_ox_ cofactor was additionally treated with potassium ferricyanide, absorption changes by FAD were minor, despite a slightly diminished absorption at 440 nm, which may be attributed to variations between crystals. Photoreduction with DTT generated the typical absorption pattern of the fully reduced FADH^–^ state. Overall, *in crystallo* spectroscopy provided a solid base for analyzing redox-state-dependent SFX data.

Photoreduction, involving ET and flavin reduction, is common in PCSf enzymes. While previous research has explored the structural dynamics of FAD transition in a class II DNA PL, *Mm*CPDII by TR-SFX,^36^ evidence regarding the structural transition of prokaryotic (6–4) PLs during photoactivation remains absent. Difference electron density (DED) maps revealed significant changes around the FAD cofactor and the [4Fe-4S] cluster in the FADH^–^ (Figure 2C) and FAD_ox_/[4Fe-4S]^3+^ states (Figure 2D), respectively. FADH^–^ exhibited substantial structural changes near FAD, indicating its transition to a fully reduced state upon illumination. Minor disparities were observed in [4Fe-4S] of FADH^–^, suggesting potential involvement in photoreduction.

In the oxidized state of *Cc*(6–4)PL, the [4Fe-4S] cluster adopts its characteristic rhomboid geometry, which is identical in the synchrotron and XFEL structures (Figure S5B). Interestingly, the superoxidized state generated by K_3_FeCN_6_ shows significant structural changes for the iron–sulfur cluster according to DED maps (Figure 2D). These changes upon oxidation of a prokaryotic (6–4) PL with iron–sulfur cluster directly affect its protein environment as discussed below.

### The Cofactor Binding Sites of *Cc*(6–4)PL

**Figure 3:**
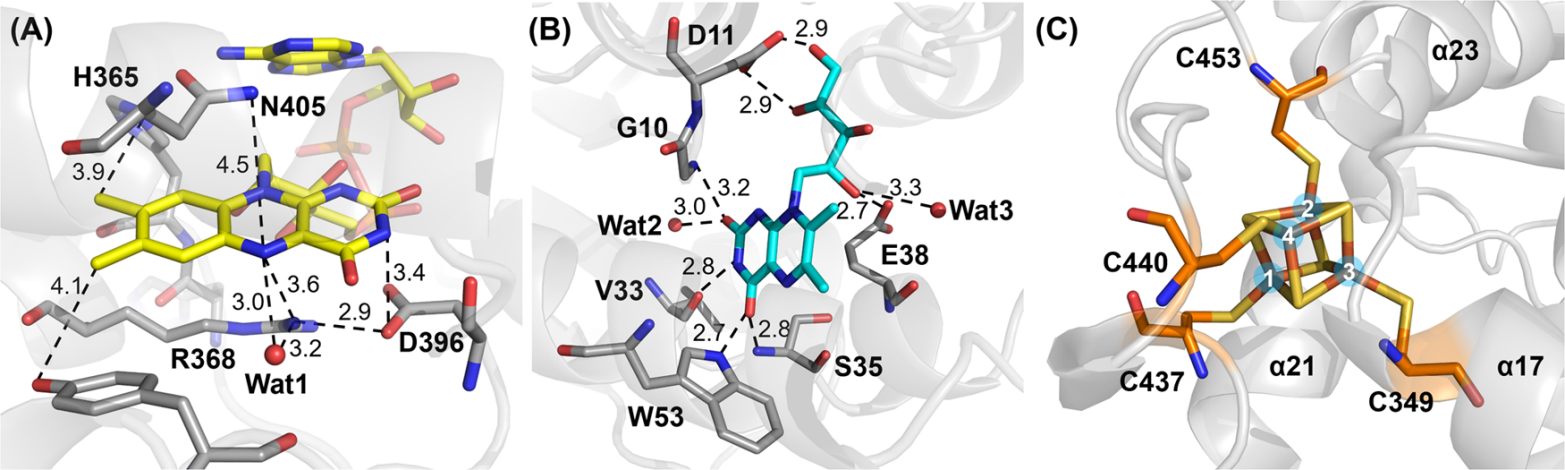
Cofactors’ interaction sites within the damage-free structure of oxidized *Cc*(6–4)PL. (A) FAD binding pocket with surrounding residues (gray sticks) and waters (red spheres). A water molecule, Wat1, forms a hydrogen bond with the N5 atom of the isoalloxazine ring; corresponding atom–atom distances are given in Angstrom (Å). (B) DLZ binding site, with nearby residues (gray) and water molecules (red), which are involved in hydrogen-bonding networks between the chromophore and the protein. (C) Binding site for the [4Fe-4S]^2+^ cluster in its C-terminal roof-like subdomain. The conserved four cysteines are shown as stick models as well as the [4Fe-4S] cluster with its numbered iron atoms.

In the context of cofactor interactions, the damage-free structures reveal FAD’s binding arrangement with its isoalloxazine moiety engaging key amino acid residues such as N405, R368, and D396. Notably, a crystallographic water molecule (Wat1) forms a hydrogen bond with the isoalloxazine N5 atom, a characteristic feature of prokaryotic (6–4) PLs, which sets them aside from other PCSf subfamilies (Figure 3A). This interaction suggests that Wat1 serves as a proton donor to N5 for converting the FAD^•–^ radical to FADH^•^ after photoreduction. These interactions highlight the conserved nature of cofactor coordination in prokaryotic (6–4) PL and underscore the functional significance of solvent molecules in modulating the catalytic activity.

A secondary cofactor, DLZ, serves as an antenna chromophore due to its enhanced light absorption compared to FAD for improving the latter’s catalytic efficacy. Structural analysis reveals an intricate DLZ binding pocket with interactions involving several key residues (Figure 3B). DLZ’s aromatic ring forms hydrogen bonds and packing interactions with S35, W53, and V33. The ribityl moiety of DLZ interacts with E38 and D11, stabilizing DLZ within the pocket. Crystallographic water molecules (Wat2 and Wat3) are part of the interaction network and contribute to the pocket stability.

The [4Fe-4S] cluster within the roof-like subdomain of *Cc*(6–4)PL (Figure 3C) is covalently linked to four conserved cysteine residues, C349, C437, C440, and C453. These cysteines maintain cluster and protein integrity, as mutants affecting the binding of the iron–sulfur cluster failed to form soluble protein,^49^ emphasizing its critical role. The Fe1 and Fe4 metal ions of the cluster being coordinated to C437 and C440, respectively, are close to the protein surface and may hence be routes for electron transfer or enzymatic catalysis.

### Structural Changes in the FADH^–^ State of *Cc*(6–4)PL

**Figure 4:**
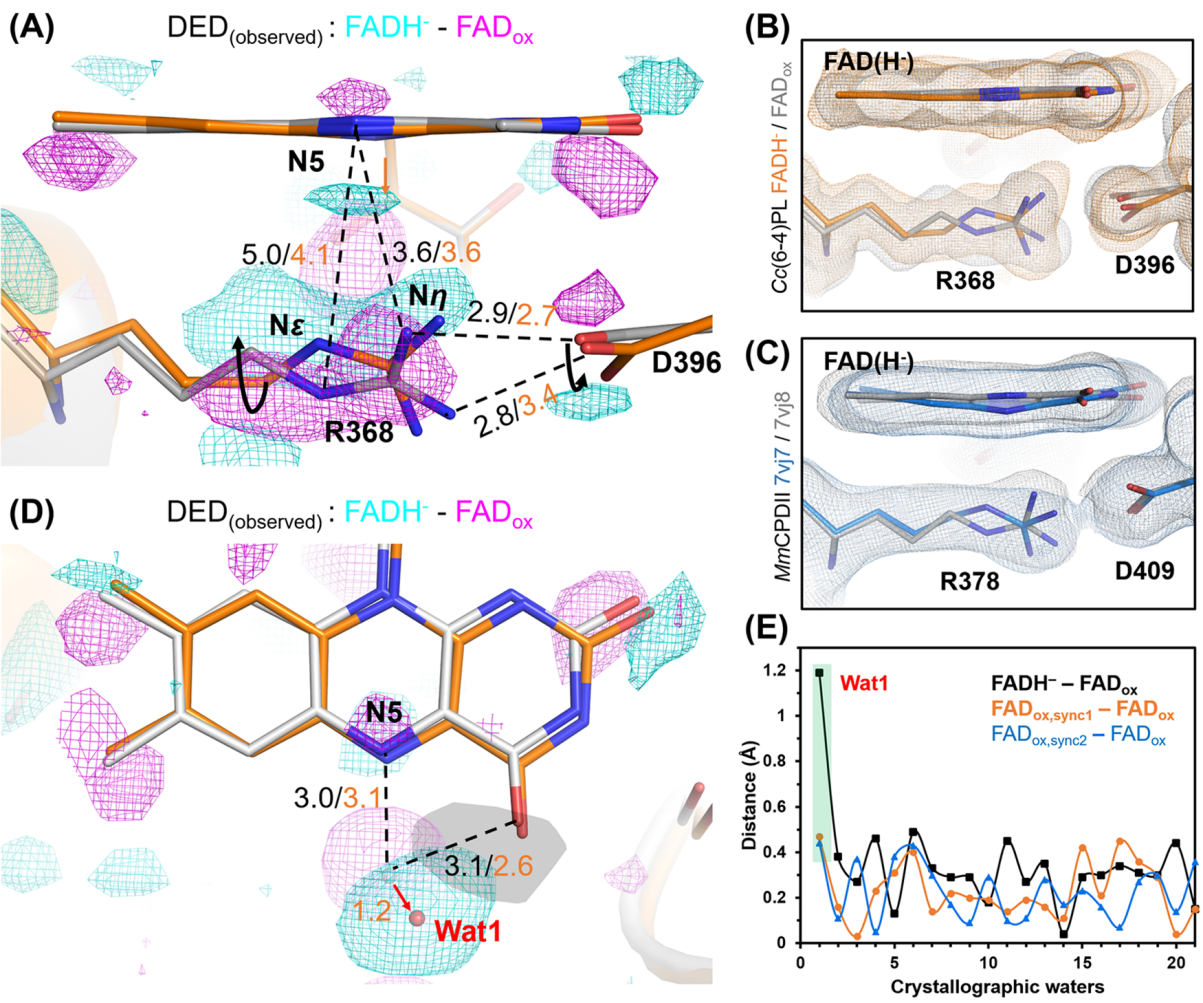
Structural changes between dark-adapted FAD_ox_ (gray) and fully reduced FADH^–^ (orange) states of *Cc*(6–4)PL. To highlight the experimental significance of the observed differences, 3.5σ contoured DED maps are shown in cyan for positive peaks and magenta for negative ones. Distances (Å) for the FAD_ox_ and FADH^–^ states are shown in black and orange, respectively. The arrows indicate movements between FAD_ox_ and FADH^–^ states. (A) Local changes of FAD, R368, and D396 moieties (stick models) in the FADH^–^ state. The FAD’s isoalloxazine is only slightly bent according to flavin real space correlation coefficient refinement.^41^ The ρC and ρN dihedral angles of the isoalloxazine group were calculated to 5.1 and 6.8°, respectively.^36^ (B) SigmaA-weighted 2*m*F_ext_–*DF*_calc_ electron density maps were contoured at the 1.0σ level. Densities near the isoalloxazine of FAD_ox_ and FADH^–^ are shown as gray and orange mesh, respectively. (C) For comparison, the conformational change at the isoalloxazine of the FAD_ox_ and FADH^–^ states is shown for the class II PL *Mm*CPDII (7vj8 and 7vj7). SigmaA-weighted 2*m*F_ext_–*DF*_calc_ electron density maps (contouring level 1.0σ) are shown in gray and blue mesh, respectively. (D) Positional movement of Wat1 due to photoreduction to FADH^–^. (E) 21 water molecules occur within a radius of 10 Å from the FAD cofactor, which are completely excluded from bulk solvent access. The plot shows their relative displacements upon the transition from the FAD_ox_ to the FADH^–^ state. Only for Wat1 is a significant shift found in the FADH^–^ state (black, highlighted by green rectangle). For comparison, displacements are also shown for the two synchrotron-based data sets relative to the SFX data set of the FAD_ox_ state (orange and blue, details in Figure S6).

In our study, we generated the FADH^–^ state in the presence of the reducing agent DTT. Upon illumination, structural changes occurred in the FAD binding site compared to the FAD_ox_ state (Figure 4A). Notably, the DED maps reveal a pronounced positive peak above the R368 Nε and Nη atoms, indicating movement of the guanidinium group toward the FAD’s N5 atom. Simultaneously, the isoalloxazine moiety of FAD exhibited a slight bending motion, resulting in a shorter distance between the FAD N5 and R368 Nε atoms. Additionally, the geometry of the bifurcated salt bridge D396-R368 altered due to swiveling motions of both side chains (Figure 4B).

These findings align with our previous investigations, particularly a study that utilized TR-SFX to elucidate the structural dynamics of *Mm*CPDII photoreduction.^36^ This study demonstrated concurrent movement of the R378 Nε moiety, the residue corresponding to R368 in *Cc*(6–4)PL, toward the N5 atom of FAD and bending of the isoalloxazine ring during the transition from the oxidized to reduced state (Figure 4C).

Like in the FAD_ox_ state, the conserved water molecule, Wat1, is positioned within hydrogen-bonding distance of the N5 atom and the carbonyl group (O4) of the FAD’s isoalloxazine moiety (Figure 3A),^16,17^ but underwent a notable shift by 1.2 Å upon photoreduction of *Cc*(6–4)PL toward the FADH^–^ state (Figure 4D). This displacement caused an approach by 0.5 Å of Wat1 toward the isoalloxazine O4 atom. Notably, in the synchrotron-derived *Cc*(6–4)PL structures of the FAD_ox_ state, Wat1 is displaced into the same direction, albeit by only <0.5 Å (Figures 4E and S6). This may be a consequence of partial FAD reduction due to radiation damage by synchrotron X-ray irradiation as observed before in class I and II PLs.^9,50,51^ DED peaks around the isoalloxazine moiety (Figure 4A, ρC = 5.1°, ρN = 6.8°) indicate subtle, but significant butterfly-like bending of the isoalloxazine upon photoreduction of *Cc*(6–4)PL to the FADH^–^ state. The observed Wat1 movement upon photoreduction can be hence caused by later protonation of the N5 nitrogen or the isoalloxazine’s conformational change.

**Figure 5:**
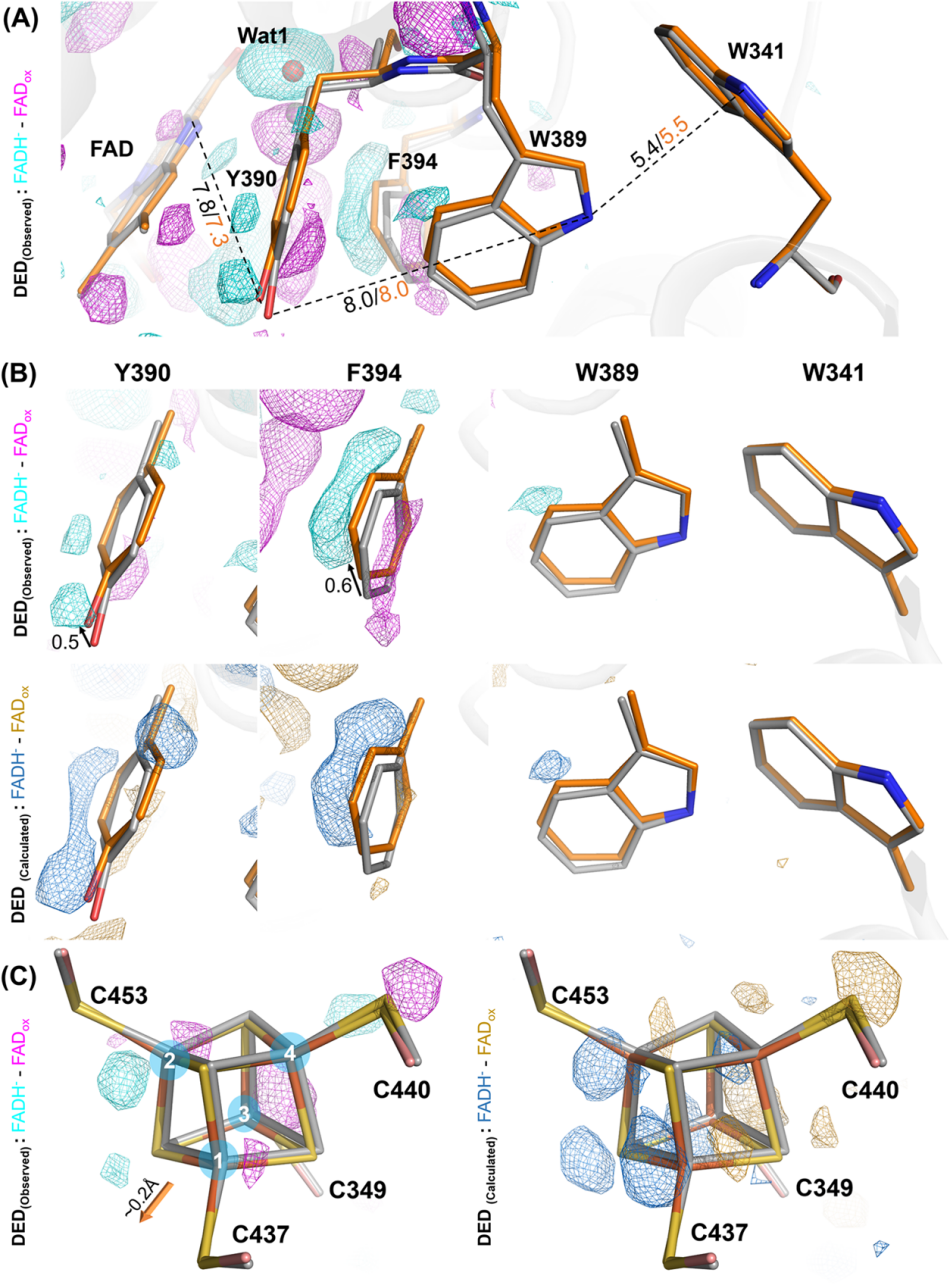
Structural changes in ET pathway and iron–sulfur cluster between dark-adapted and fully reduced states. FADH^–^ state of *Cc*(6–4)PL (orange) is shown in comparison of FAD_ox_ (gray). To highlight the experimental significance of the observed differences, 3.5σ contoured DED maps are shown in cyan for positive peaks and magenta for negative ones. For calculated differences, 3.5σ contoured DED maps are shown in blue for positive peaks and in gold for negative ones. The arrows indicate movement of residue between FAD_ox_ and FADH^–^. (A) Overview of structure changes in the intramolecular electron transfer chain. The residues Y390 and F394 indicate a shift toward the FAD, thus shortening the distance to the FAD’s isoalloxazine moiety. (B) Detailed views for each residue in the ET pathway. (C) Structure changes in [4Fe-4S]. The iron–sulfur cluster shows a slight displacement in the FADH^–^ state whose direction is indicated by an arrow (orange). The map correlation between observed DED maps and calculated ones is shown to be high. The map correlation value can be found in Table S2.

Within other subfamilies of the PCSf, initial light-driven ET events are known to proceed in rates of less than a nanosecond.^11,52^ However, prokaryotic (6–4) PLs lack the conserved tryptophan triad, which is common in almost all other PCSf subfamilies. Instead, they harbor a unique pair of proximal tyrosines or phenylalanines next to the FAD’s isoalloxazine moiety (*Cc*(6–4)PL: Y390/F394). Beyond these residues, the intramolecular ET pathway is continued by two conserved tryptophans, medial W389 and distal, surface-exposed tryptophan W341 (Figure 5A). Although many prokaryotic (6–4) PLs harbor at least one redox-active tyrosine for the proximal aromatic pair, e.g., as Yx_3_Y motif in *Vc*(6–4)PL from *Vibrio cholerae* (Figure S7)^33^ or PhrB from *Agrobacterium tumefaciens*,^17^ others have an Fx_3_F motif like the (6–4) photolyase from *Methanothermobacter marburgensis* (Uniprot: D9PUQ6). Likewise, a Yx_3_Y → Fx_3_F conversion in *Rs*(6–4), the (6–4) photolyase from *Rhodobacter sphaeroides*, still allows photoreduction in contrast to mutations of the medial and distal tryptophans.^16^ Although redox potentials for the proximal aromatic residues of ∼1.6 V for *E*°(F^•+^/F) and ∼1.35 V for *E*(YH^•+^/YH) are high compared to ∼1.07 V for *E*°(WH^•+^/WH) of the medial tryptophan, they may still allow direct reduction of the photoexcited FAD in the S1 state with its *E*°(FAD*/FAD^•–^) of ∼2.45 V and subsequent ET from the medial tryptophan. Comparing now the structures of *Cc*(6–4)PL for its FADH^–^ and FAD_ox_ states, we observe no conformational changes for medial and distal tryptophans W341 and W389. However, in the FADH^–^ state, the proximal Y390 and F394 move with their side chains toward the FAD moiety, decreasing the interatomic distances by 0.5 Å and 0.6 Å, respectively (Figure 5A,5B). The change of Y390 is accompanied by tilting its peptide group by ∼11° into the helix axis (φ_439,ox_/Ψ_439,ox_: −64.0/–28.2°, φ_439,red_/Ψ_439,red_: −62.1/–38.8°). Accordingly, the carbonyl group that forms a hydrogen bond to Wat1 in both states increases the helicity (average α-helical ϕ/Ψ: −62/–41°)^53^ of the C-terminal end of helix α18 in the reduced form.

The side chain of K48 is almost halfway located between the DLZ antenna in the N-terminal domain and the FAD’s isoalloxazine. Upon transition from the FAD_ox_ to the FADH^–^ state it loses its hydrogen bond to the peptide linking Y390 with M391 while shortening its H-bond to the peptide of A397 (3.0 vs 2.3 Å) (Figure S8A). Although the displacement of the K48 side chain may be dictated by the changed electrostatics around the isoalloxazine moiety and Wat1 movement, this residue participates in the stabilization of DLZ binding. A K48A mutation results in a partial loss of the DLZ antenna, which can be observed through UV/vis spectroscopy (Figure S8C). This is further supported by the structure of the K48A mutant, where the DLZ antenna appears to be only partially occupied (Figure S8B). Overall, the shift of water molecule Wat1 upon photoreduction affects the local environment, including the ET pathway and the binding site of the antenna chromophore.

Regarding iron–sulfur clusters, their role as redox-active cofactors is well-known in many biological processes, but their specific function within prokaryotic (6–4) PLs is still poorly understood. The [4Fe-4S] clusters exhibit different oxidation states, with each serving specific functions. [4Fe-4S]^1+/2+^ states act as electron donors, while [4Fe-4S]^2+/3+^ states prevail in high-energy proteins. The highly reducing [4Fe-4S]^0^ state occurs in nitrogenases, but evidence for involvement of a fully oxidized [4Fe-4S]^4+^ cluster remains elusive.^54,55^ In PhrB, light-induced responses of the iron–sulfur cluster have been traced by serial Laue diffraction methodology and assigned to changes of the redox state and mixed valence layers due to spin coupling.^55^ Interestingly, our structural analysis and the DED peaks show only minor changes for the [4Fe-4S] cluster itself upon light illumination (Figure 5C).

These can be modeled as a slight displacement of the cluster (∼0.2 Å) along the Fe4-to-Fe1 vector. Otherwise, our damage-free structures show no significant changes of the geometry of the iron–sulfur cluster in its different redox states (Table S3). Given that EPR experiments indicate an unchanged [4Fe-4S]^2+^ redox species for the *Cc*(6–4)PL FADH^–^ state, this damage-free structure does not support permanent redox status changes of the [4Fe-4S] cluster concomitant with photoreduction at the FAD cofactor site.

### Transition to [4Fe-4S]^3+^ State Causes Dynamic Loosening of an Iron–Sulfur Bond

**Figure 6:**
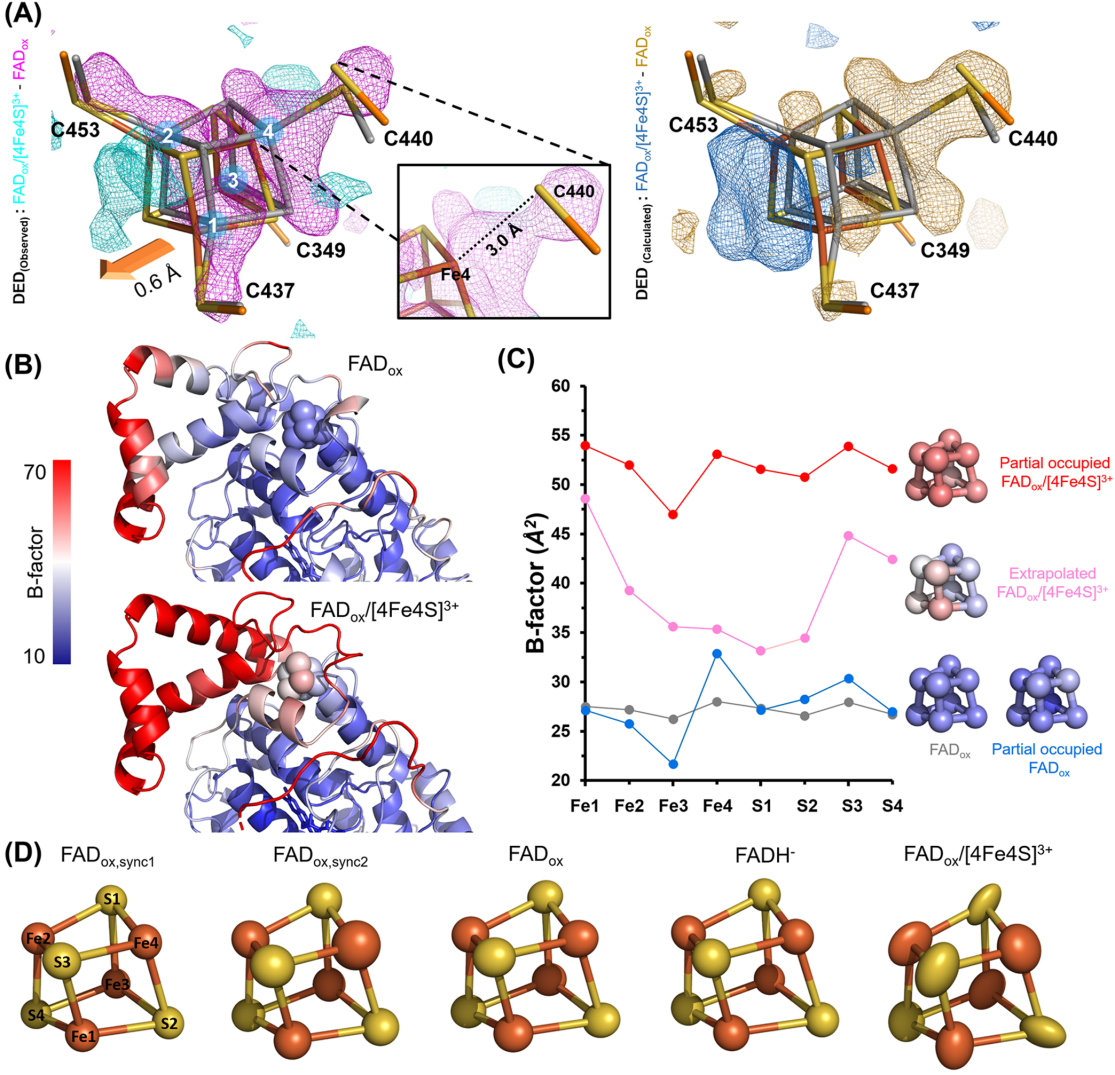
Structural changes in iron–sulfur cluster between FAD_ox_ and FAD_ox_/[4Fe-4S]^3+^ states. (A) Structural changes in the [4Fe-4S] cluster upon its oxidation. The [4Fe-4S] cluster shifts by ∼0.6 Å toward the solvent (arrow, orange), whereas the components of [4Fe-4S] exhibit complete covering of negative density. The FAD_ox_/[4Fe-4S]^3+^ state of *Cc*(6–4)PL (orange) is shown in comparison of FAD_ox_ (gray). For highlighting the significance of observed differences, 3.5σ contoured DED maps are shown in cyan and magenta for positive/negative peaks. For calculated differences, 3.5σ contoured DED maps are likewise shown in blue and gold for positive/negative peaks. The map correlation between observed DED maps (left) and calculated ones (right) is shown to be high; the map correlation value is listed in Table S2. The inlay shows atom–atom distances in Angstrom. (B) *B*-factor distribution around the roof-like domain. Oxidation of the [4Fe-4S] cluster to the +3 state causes increased *B*-factors for regions of the roof-like domain, which are well ordered in the +2 state. (C) *B*-factor plots and CPK models of the [4Fe-4S] cluster in its +2 and +3 states. Oxidation toward the FAD_ox_/[4Fe-4S]^3+^ state (pink, from extrapolated data) causes increased thermal mobility when compared with the FAD_ox_ state (gray). Increased disorder of the [4Fe-4S]^3+^ cluster can be also delineated from B-factor distributions, when performing instead partial-occupancy refinement of both the FAD_ox_/[4Fe-4S]^2+^ (66% occupancy, blue) and FAD_ox_/[4Fe-4S]^3+^ (34%, red) states against nonextrapolated FAD_ox_/[4Fe-4S]^3+^ SFX data. (D) Anisotropic B-factor refinement of the [4Fe-4S] cluster. The anisotropic thermal motion of each atom on [4Fe-4S] at redox states was depicted as ellipsoids.

In aerobic organisms, high levels of oxygen can lead to toxicity by generating reactive oxygen species (ROS) such as superoxide and hydrogen peroxide. These ROS can harm essential enzymes, particularly those containing [4Fe-4S] clusters, disrupting vital metabolic pathways like the TCA cycle.^56^ Fe–S clusters are susceptible to oxidation due to their exposure to solvent, allowing direct interaction with ROS. When Fe–S clusters undergo oxidation, they become unstable and prone to decomposition. Superoxide or hydrogen peroxide triggers the oxidation of the [4Fe-4S] cluster, resulting in a chemical conversion from the stable [4Fe-4S]^2+^ form to an unstable [4Fe-4S]^3+^ state. This alteration can cause the release of iron ions from the cluster, transforming it into a [3Fe-4S]^1+^ configuration.^54^ Our structural analysis reveals significant structural changes within the [4Fe-4S] cluster, as evident from the DED maps (Figure 6A). The entire [4Fe-4S] cluster shows negative density, that extends to the covalent bonds made from the cysteines to the irons of the cluster, indicating substantial structural changes. The DED maps show a shift of the whole [4Fe-4S] cluster by 0.6 Å toward the solvent-exposed side of the roof-like subdomain. Notably, the distance between the C440 Sγ and Fe4 atoms increases from 2.3 to 3.0 Å (Figure 6A, inlay), indicating bond breakage upon oxidation of the iron–sulfur cluster to the [4Fe-4S]^3+^ state (Figure 6A). Partial loss of attachment of the [4Fe-4S] cluster to the roof-like subdomain is not only concomitant to the 0.6 Å displacement of the [4Fe-4S] cluster along the Fe3-to-Fe1 and S2-to-Fe1 vectors but also causes increased mobility of the subdomain as judged from elevated thermal B-factors of the subdomain’s main chain (Figure 6B).

Likewise, the atoms of the [4Fe-4S] cluster exhibit significantly elevated B-factors, particularly Fe1, Fe2, S3, and S4 (Figure 6B,C). However, there is no loss of occupancy for individual atoms that would result from partial decomposition of the iron–sulfur cluster. At least for the refined structure from extrapolated FAD_ox_/[4Fe-4S]^3+^ data, increased mobility of the [4Fe-4S] cluster is accompanied by directional atomic displacements (Figure 6D). In the superoxidized FAD_ox_/[4Fe-4S]^3+^ state, the remarkable thermal anisotropy within the iron–sulfur cluster as compared to other redox states may indicate a strong dynamic or positional disorder due to the breakage of the Fe4–C440Sγ bond. However, being aware that data extrapolation can cause systematic changes in B-factor distributions,^57^ we reanalyzed the thermal B-factor distributions of the FAD_ox_/[4Fe-4S]^3+^ and FADH^–^ states by partial-occupancy refinement of our structural models against raw data sets (see Supporting Information). This approach reproduced for the protein part the B-factor distributions with Pearson correlation coefficients of 92 and 90% for the FAD_ox_/[4Fe-4S]^3+^ and FADH^–^ states, respectively. This includes especially the elevated thermal mobilities of the roof-like subdomain in the FAD_ox_/[4Fe-4S]^3+^ state (Figure S9). Overall, redox transition from [4Fe-4S]^2+^ to [4Fe-4S]^3+^ upon oxidation triggers structural instability of the roof-like subdomain and significant [4Fe-4S]^3+^ cluster shifting due to bond breakage of the Fe4 and C440 Sγ atoms.

## Discussion

In our study, we explored the overall structural features of the *Cc*(6–4)PL and highlighted structural changes in the catalytic FAD and [4Fe-4S] cluster across different redox states using SFX. Since FAD plays a central role in PCSf members, one might expect a unified mechanism to regulate light-dependent flavin redox chemistry. In free solution, butterfly bending of the isoalloxazine moiety affects FAD dynamics in its anionic or neutral state.^10,58^ However, the actual bending observed for PLs in their catalytic FADH^–^ state is variable. In the FADH^–^ state of *Cc*(6–4)PL, we identified only a slight butterfly-like bending of the isoalloxazine moiety upon photoreduction. With ρC and ρN angles of 5.1 and 6.8° (FAD_ox_ state: 0.2/0.1°), it is considerably less than in the steady-state structures of *Mm*CPDII^36^ (14.3/14.5°) and the class I CPD PL from *Anacystis nidulans*, *An*CPDI, at least 8.8/9.5° as obtained from synchrotron data.^50^ Accordingly, prokaryotic (6–4) PLs lack the stabilization of the FADH^–^ state of some of the other PL subfamilies, where the N5 and N10 atoms of the FAD’s isoalloxazine show an increased sp^3^-like nature. This destabilizing effect of the protein matrix on the FADH^–^ cofactor may not only lower the redox potential of the ground state,^58,59^ but also that of the photoexcited state, which causes electron transfer to the (6–4)PP lesion for DNA repair. Notably, protein matrix effects on the conformation of flavin chromophores can also affect the transient structures of flavins, which evolve shortly after ET. During the FAD_ox_ → FAD^–•^ photoreduction, *Mm*CPDII adopts a butterfly-like angle of 18.4°—ρC/ρN are 20.3/18.8° after 125 μs^36^ —whereas the eukaryotic (6–4) PL *Dm*(6–4) achieves only 2.4° after 100 μs (0.8/1.4°, Table S4).^60^

**Figure 7:**
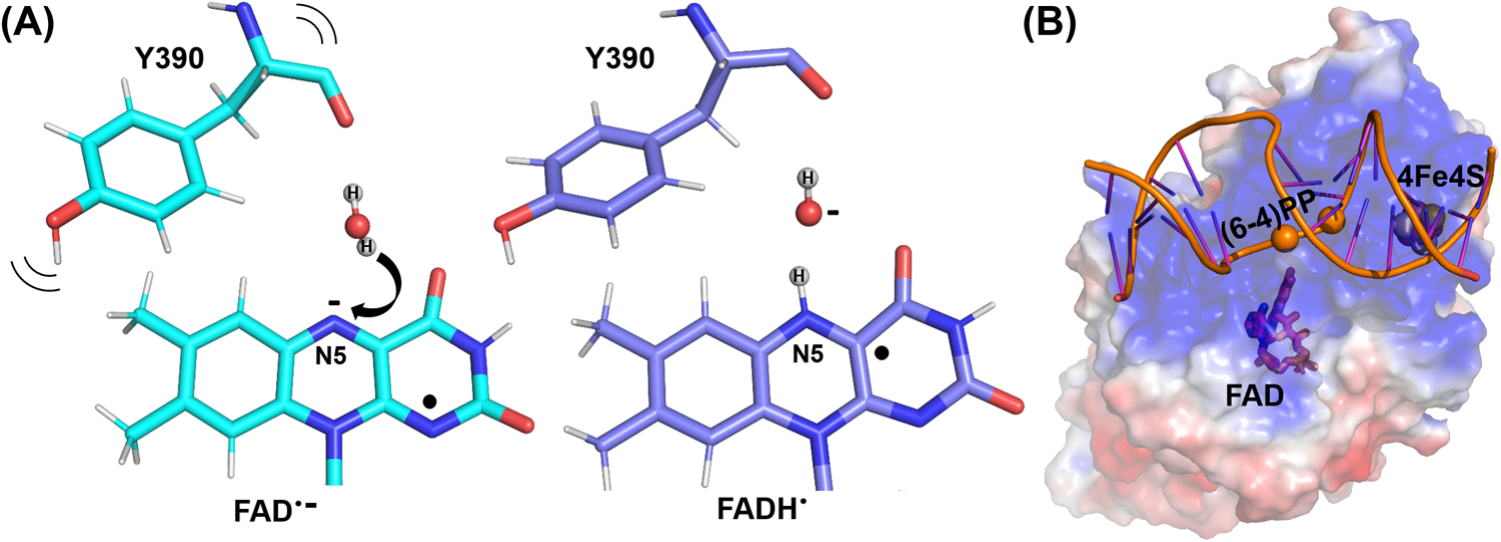
Models of the *Cc*(6–4)PL action. (A) Proton transfer from water to FAD^•–^ during photoreduction of prokaryotic (6–4) PL. Hydroxide formation triggers slight conformational rearrangements nearby to allow for reprotonation. (B) Electrostatic surface of *Cc*(6–4)PL superimposed to an Alphafold3 model^62^ of a 15-meric duplex DNA with *Cc*(6–4)PL. To get a docked AF3 pose, the sites of the (6–4)PP lesion were treated as abasic sites (spheres). For comparison, the [4Fe-4S] cluster is depicted as a CPK model and the FAD chromophore as sticks (details in Figure S12).

Within the PCSf family, the characteristic Arg-Asp salt bridge and the asparagine next to the FAD’s N5 atom were supposed to form a redox sensor triad (*Mm*CPDII: N403,^36^*Dm*6–4: N403^60^). Conversely, in prokaryotic (6–4) PL, a single water molecule (Wat1) is strategically positioned near the substituted asparagine residue to establish hydrogen bonds with the N5 nitrogen of FAD. Previous investigations have elucidated the role of arginine as a stabilizing element during ET-driven formation of the FAD^•–^ radical, while the asparagine is involved in later protonation events and contributes to the stabilization of FADH^•^.^61^ In the fully reduced state of *Mm*CPDII, hydrogen bonds formed between FAD N5–N403 Oδ and FAD N5–R378 Nε collectively contribute to the stabilization of FADH^–^. In the oxidized and fully reduced redox states of *Cc*(6–4)PL, Wat1 retains its hydrogen bond with N5 and exhibits even closer proximity to the carbonyl group (O4) of the isoalloxazine moiety in the FADH^–^ state. Indicating its role in modulating the cofactor’s redox state within the active site environment, the repositioning of Wat1 apparently stabilizes the FADH^–^ state and affects the C-terminal end of helix α18 with its proximal aromatic ET residue Y390. Although our data do not capture the semiquinoid steady state (FADH^•^), R368 in *Cc*(6–4)PL likely stabilizes the transient FAD^•–^ radical during photoreduction as R378 of the same conserved salt bridge is doing in *Mm*CPDII.^36^ However, the protonation pathway responsible for the transition from the FAD^•–^ state to FADH^•^ remains elusive within FeS-BCPs. Asn405 is too distant from the N5 nitrogen to fulfill the role of a proton donor as claimed for the conserved asparagine N395 in the cryptochrome *Cra*CRY.^61^ Obviously, Wat1 that is associated in all redox states with the N5 nitrogen is the prime candidate as a proton donor during the FAD^•–^ → FADH^•^ transition (Figure 7A).

Reprotonation of a thus-formed hydroxide anion next to the FADH^•^ chromophore requires the opening of a transient protonation pathway. In *Cc*(6–4)PL, such a pathway may include nearby residues E402 and D387, whose carboxyl groups are apparently hydrogen-bonded via a shared proton (Figure S10). Notably, the significant movement of K48’s side chain halfway between the DLZ and the FAD chromophore (Figure S8A), previously unreported, suggests further stabilization of the FADH^–^ state due to electrostatic interactions. Although this residue does not directly interact with either cofactor, i.e., DLZ or FAD, it also contributes to the binding of the DLZ antenna, as this antenna is partially lost in the K48A mutant (Figure S8C). In the 1.5 Å structure of the K48A, we find only an occupancy of ∼50% for the antenna.

The [4Fe-4S] cluster is well-known for its indispensable role in facilitating electron transfer processes such as those in photosynthesis and respiration. Furthermore, many processes, such as DNA replication and DNA repair, depend on enzymes and DNA-binding proteins, which harbor [4Fe-4S] clusters. Besides a merely structural role, these [4Fe-4S] clusters were claimed to be involved in charge-transfer processes along DNA (DNA-CT).^63^ In prokaryotic (6–4) PLs, the large distance of ∼18 Å and the large gap between the potentials of the inbuilt HIPIP-like [4Fe-4S]^2+^ cluster (∼0.3 V) and the photoexcited flavin chromophore are negligible for direct ET by being in the inverted regime of the Marcus theory to compete with the inbuilt ET pathway using a chain of aromatic side chains. Given this, the function of the built-in [4Fe-4S] cluster of prokaryotic (6–4) PLs is still elusive. In *Cc*(6–4)PL, we examined its [4Fe-4S] cluster in its +2 and +3 states which can be also adopted by other orthologs like PhrB and *Rs*(6–4). Indeed, EPR spectra of the [4Fe-4S]^3+^ cluster in prokaryotic (6–4) PLs show X-band spectra, which are reminiscent, but not identical to those of the [4Fe-4S]^3+^ cluster of high-potential iron–sulfur proteins. The lack of a negative amplitude as in HiPIP X-band spectra and the appearance of a small positive signal at *g* = 2.002 may indicate a contribution by a thiyl radical. In free solution, thiyl radicals of cysteine appear within the *g*-value range of 2.03 to 2.002.^64^ Our damage-free XFEL structure of *Cc*(6–4)PL that has been oxidized to the [4Fe-4S]^3+^ state shows interestingly an apparent lengthening of the iron–sulfur bond linking the Fe4 ion with C440. The observed increase from the canonical 2.3 to 3.0 Å can be interpreted either as a bond breakage or, more likely, a result of dynamic interchange between coordinated and noncoordinated C440. The latter is corroborated by an occupancy of the [4Fe-4S]^3+^ state of ∼33% in the SFX structure that is about 5-fold higher than observable by UV–vis spectroscopy. The dynamic interchange suggested by this discrepancy for occupancy also explains the EPR signature of *Cc*(6–4)PL in its [4Fe-4S]^3+^ state, as the signals for the [4Fe-4S]^3+^ cluster and the putative thiyl radical do not show any spin–spin interaction. Accordingly, we suggest that the cluster either coordinates to all four cysteines when in its +3 state or releases C440 as a thiyl in the +2 state with only three cysteine ligands remaining. Such a reversible cleavage of the Fe–S bond has been recently implied for [4Fe-4S] clusters in synthetic complexes and a metalloprotein^65^ due to their capability to rapidly exchange Fe^2+^ ions with their environment. Interestingly, dynamic loosening is observed only for the single Fe–S bond linking Fe4 and C440. For Fe atoms Fe2 and Fe3, this is understandable as these irons coordinate to the surface-occluded residues C349 and C453. In contrast, the other two cysteines, C437 and C440, are consecutive parts of a surface-exposed loop linking helices α22 and α23. Other effects of the protein matrix may determine the preferential cleavage of the Fe–S bond made by C440. In HIPIP, the region C43–C46 mimics closely the stretch C437–C440 of *Cc*(6–4)PL^47,66^ (Figure S11). Here, C43 and C46 coordinate the redox-active subcluster with its two irons and μ-bridging sulfur atoms toward external electron donors via hydrogen bonds made by C46. In *Cc*(6–4) PL, these H-bonds are preserved, although in a different structural context, as the C440 Sγ atom makes hydrogen bonds to two donors, A348N (3.6 Å) and Wat105 (3.9 Å).

A prior study on PhrB combined serial crystallography with 5 μs 24-bunch Laue X-ray pulses from a synchrotron source and continuous laser illumination at 405 and 450 nm, producing detailed difference electron density (DED) maps at the embedded [4Fe-4S] cluster.^55^ However, with Laue X-ray fluxes reaching 10^11^–10^12^ photons per exposure at the sample,^67^ radiation damage to the [4Fe-4S] cluster is unavoidable. Using singular-value decomposition (SVD) analysis, the authors interpreted the DED maps of the photoexcited [4Fe-4S] cluster as evidence of complex changes in its redox state and even spin coupling between valence layers from the DED maps of the photoexcited [4Fe-4S] cluster. By contrast, our robust DED signals for chemically defined states of *Cc*(6–4) can be explained by subtle shifts of the entire iron–sulfur cluster. Unlike the Laue diffraction method, SFX at X-ray free-electron lasers is now widely accepted as an effective method for avoiding X-ray-induced artifacts, owing to the “diffraction-before-destruction” effect,^68^ especially in systems with redox-sensitive metalloclusters.^69^ Ultimately, the prominent signals observed near the FeS-cluster likely arise from displacements and conformational changes in the cluster rather than requiring interpretations involving spin-coupled states or electron redistribution.

In contrast, the FADH^–^ structure reveals only minor changes in the geometry of the [4Fe-4S]^3+^ cluster when compared to the [4Fe-4S]^2+^ state. Other redox states for the iron–sulfur cluster such as a reduced [4Fe-4S]^1+^ state as claimed before by singular-value decompositions of Laue data from PhrB^55^ are not observed for our FADH^–^ state, neither structurally nor by an EPR signature. Accordingly, there is no indication of direct ET between the FAD and iron–sulfur cofactors in *Cc*(6–4)PL during photoreduction, a question that may be addressed via a time-resolved structural analysis. Given the continuity between the binding site of the (6–4)PP lesion and the positively charged surface of the roof-like subdomain that harbors the iron–sulfur cluster (Figures 7B and S12), it is plausible that the iron–sulfur cluster may potentially affect DNA repair, e.g., by DNA-CT. The model of the *Cc*(6–4)PL·DNA complex positions the 3′ arm of the duplex DNA next to the (6–4)PP lesion in such a way that it forms numerous electrostatic interactions with the [4Fe-4S] cluster harboring roof-like subdomain. Oxidation of the [4Fe-4S]^2+^ cluster, e.g., by charge transfer to an electron hole within duplex DNA, may destabilize the loop associated with C440 and affect the affinity to DNA. Such redox-dependent affinity changes to DNA were observed before for the p58 subunit of primase,^70^ which is structurally directly related to the all-α-helical domain of prokaryotic (6–4) PLs.

Overall, the observation that [4Fe-4S] clusters in prokaryotic (6–4) photolyases are not inherently stably coordinated by their cysteine residues but instead undergo a dynamic exchange involving transient Fe–S bond cleavage suggests that the redox chemistry of these high-potential clusters may influence the structural dynamics of the surrounding protein matrix to a greater extent than previously considered.

## Supporting Information Available

Additional experimental details for the DNA repair assay; method for deriving and validating *B*-factor distributions from SFX data UV/vis spectra for *Cc*(6–4)PL and its cofactor contributions; and structural details of *Cc*(6–4)PL SFX structures (PDF)
