# Supplementary material for: Redox-State-Dependent Structural Changes within a Prokaryotic 6–4 Photolyase
Source: J Am Chem Soc. 2025 Apr 29;147(19):16084–98. doi: 10.1021/jacs.4c18116 (PMC12082625; doi:10.1021/jacs.4c18116)
Supplement: Supplementary file 1 — ja4c18116_si_001.pdf [file ja4c18116_si_001.pdf]

SUPPORTING INFORMATION

Redox-state dependent structural changes within a prokaryotic 6-4 photolyase

Po-Hsun Wang,[a][b] Yuhei Hosokawa,[c][d] Jessica C. Soares,[e] Hans-Joachim Emmerich,[a] Valeri Fuchs,[a] Nicolas Caramello,[f][g] Sylvain Engilberge,[f][h] Andrea Bologna,[i] Christian Joshua Rosner,[a] Mai Nakamura,[d] Mohamed Watad,[a] Fangjia Luo,[j] Shigeki Owada,[j][k] Takehiko Tosha,[k] Jungmin Kang,[k] Kensuke Tono,[j][k] Yoshitaka Bessho,[b][l] Eriko Nango,[k][m] Antonio J. Pierik,[e] Antoine Royant,[f][h] Ming-Daw Tsai,[b] Junpei Yamamoto,[d] Manuel Maestre-Reyna,*[b][c] Lars-Oliver Essen*[a]

[a] Department of Chemistry
Philipps University Marburg
Hans-Meerwein Strasse 4, Marburg 35032, Germany
[b] Institute of Biological Chemistry
Academia Sinica
128 Academia Rd. Sec. 2, Nankang, Taipei, 115, Taiwan
[c] Department of Chemistry
National Taiwan University
1, Roosevelt Rd. Sec. 4, Taipei 106, Taiwan
[d] Division of Chemistry
Graduate School of Engineering Science, Osaka University
1-3 Machikaneyama, Toyonaka, Osaka 560-8531, Japan
[e] Biochemistry, Faculty of Chemistry
University of Kaiserslautern
Kaiserslautern D-67663, Germany
[f] European Synchrotron Radiation Facility
38043 Grenoble, France
[g] Hamburg Centre for Ultrafast Imaging
University of Hamburg
22761 Hamburg, Germany
[h] Univ. Grenoble Alpes, CNRS, CEA Institut de Biologie Structurale
38044 Grenoble, France
[i] Department of Science and Technology
University of Sannio
Via Francesco de Sanctis, snc, 82100, Benevento, Italy
[j] Japan Synchrotron Radiation Research Institute
1-1-1 Kouto, Sayo-cho, Sayo-gun, Hyogo 679-5198, Japan
[k] RIKEN SPring-8 Center
1-1-1 Kouto, Sayo-cho, Sayo-gun, Hyogo 679-5148, Japan
[l] Graduate School of Agricultural and Life Sciences
University of Tokyo
1-1-1 Yayoi, Bunkyo, Tokyo 113-8657, Japan.
[m] Institute of Multidisciplinary Research for Advanced Materials, Tohoku University

2-1-43 1 Katahira, Aoba-ku, Sendai 980-8577, Japan.

CONTENT

Supporting Methods 3
In vitro DNA repair assay 3
Determining B-factors of partially occupied structures 3
Supporting Figures 4
S1. Purification of size exclusion chromatography (SEC) and crystallization of *Cc*(6-4)PL 4
S2. Estimation of redox state occupancy 5
S3. *In vitro* DNA-repair assay 6
S4. UV-Vis absorption spectroscopy of *Cc*(6-4)PL in its different redox states 7
S5. The overall oxidized *Cc*(6-4)PL structures 8
S6. The water1 movement on three oxidized states and a reduced state 9
S7. Structural superimposition of electron transfer chain in FeS-BCP members 10
S8. Structure comparison between FAD_{ox} and $FADH^{-}$ around DLZ binding pocket 11
S9 B-factor distributions of *Cc*(6-4)PL residues in its different redox states as obtained from SFX data 12
S10. Structural comparison between FAD redox states along the putative protonation pathway 13
S11. Comparison of the [4Fe-4S] clusters in *Cc*(6-4)PL and HIPIP from *Chromatium tepidum* 14
S12. The electrostatic surface potential analysis was done by APBS 15
Supporting Tables 16
Table S1. Synchrotron and SFX data statistics for steady-state structures. 16
Table S2. Map correlation values between observed and calculated DED maps on the residues 17
Table S3. The distances of atoms within iron sulfur cluster in damage-free structures 18
Table S4. State-dependent structural distortions of the FAD's isoalloxazine moiety 19
Table S5. Synchrotron data statistics for the *Cc*64PL-K48A structure 20
References 21

Supporting Methods

In vitro DNA repair assay

The 100 µM (6-4)PPs were generated with oligo$(dT)_{18}$ by using a Shimadzu RF-5301 fluorimeter through UV irradiation (260 nm). The (6-4)PPs formation causes absorption increasing at 325 nm and monitored by UV-Vis spectroscopy. After 6 hours of UV irradiation, the average number of two (6-4)PP lesions per $(dT)_{18}$ molecule was calculated by using the LAMBERT-BEER law[1], based on the difference extinction coefficient of the (6-4)PP (Δε325 nm = 6000 m^{-1} cm^{-1})[2] and the absorbance changes at 325 nm (ΔA325 nm).

In vitro DNA repair assay was carried out according to a modified protocol[3]. First, 10 µM *Cc*(6-4)PL was illuminated by a blue light LED (λ= 455 nm, 5.9 mW, distance: 4 cm) in the presence of 25 mM DTT to produce the fully reduced state in solution. Afterwards, the fully reduced *Cc*(6-4)PL was mixed with 16 µM damaged oligo-$(dT)_{18}$ in preparation for the assay. The resulting final protein concentration and DTT were 3.2 µM and 8 mM, respectively. The progress of the repair assay was determined by monitoring the change in absorbance of 325 nm. To investigate the influence of Mg^{2+} ions on the efficacy of repair, a reaction mixture consisting of 5 mM $MgCl_2$ was used for the second experiment. The same blue light LED (distance: 4 cm) was used to activate the repair reaction at 10 °C for 60 minutes.

Determining B-factors of partially occupied structures.

The B-factor is a critical parameter in structural biology, offering insights into protein dynamics, conformational flexibility, and thermos stability. It plays a pivotal role in applications ranging from predicting protein conformational disorder to guiding efforts in protein stabilization. However, describing B-factors in extrapolated structures poses significant challenges. Specifically, mean B-factors derived either from extrapolated datasets or from partial-occupancy refinements are almost linearly dependent on the inverse of the occupancy. Furthermore, the way of refining SFX structures can introduce other systematic artifacts in overall B-factor distributions, e.g. B-factor distributions from extrapolated data tend to show increased mobility for some regions, which are not found in partial-occupancy refinements[4]. To address this issue, we used our structures, the reference state FAD_{ox} and the activated one, e.g. the $FADH^-$ state from extrapolated $FADH^-$ data, and subjected them to partial occupancy refinement, here against the non-extrapolated $FADH^-$ SFX dataset (Fig. S11). In this refinement, coordinates were fixed and only B-factors and site-specific occupancies were allowed to vary[4]. For the $FADH^-$ state structure, the oxidized state was assigned an occupancy of 60%, i.e. the activated $FADH^-$ state had occupancy of 40%. For the superoxidized structure, the resting FAD_{ox} state was modeled at 66% occupancy, implying 34% for the superoxidized FAD_{ox}/$[4Fe\text{-}4S]^{3+}$ state. These alternate models were subsequently combined into a single structural file as alternative conformers, each with their respective occupancies. By comparing B-factor distributions from partial-occupancy refinements and refinements performed against extrapolated data, we can exclude that elevated B-factors for the C-terminus and roof-like subdomain of the FAD_{ox}/$[4Fe\text{-}4S]^{3+}$ state are caused by systematic errors as outlined above.

Supporting Figures

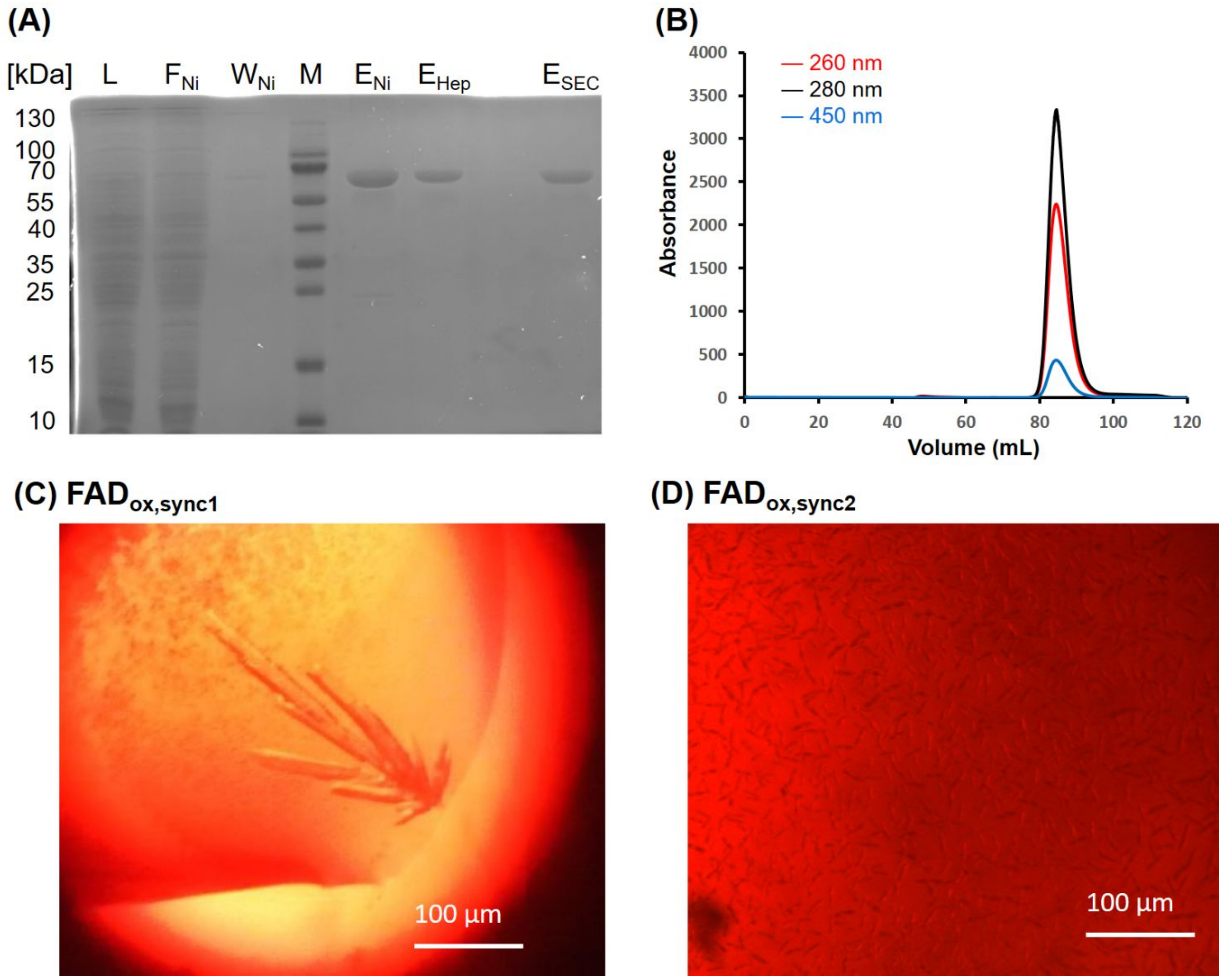

Figure S1. Purification of size exclusion chromatography (SEC) and crystallization of *Cc*(6-4)PL. (A) 12% SDS-PAGE gel of *Cc*(6-4)PL; L: lysate; F_{Ni}: flow through of nickel-NTA immobilized metal affinity chromatography (IMAC); W_{Ni}: wash fraction of IMAC; E_{Ni}: elution fraction of IMAC; E_{Hep}: elution fraction of heparin affinity chromatography; E_{SEC}: elution fraction of SEC; M: protein marker. The purity of *Cc*(6-4)PL was over 95% after final SEC. (B) The SEC chromatogram showed a single major absorbance peak corresponding to the *Cc*(6-4)PL band (calculated mass: 60.41 kDa) in the SDS-PAGE. (C) Crystals of *Cc*(6-4)PL for synchrotron- and SFX-based data recording. *Cc*(6–4)PL macro-crystals (C) and micro-crystals (D) that were used for the $FAD_{ox,sync1}$ and $FAD_{ox,sync2}$ data collection, respectively. Microcrystals of (D) were also employed for the SFX data collection.

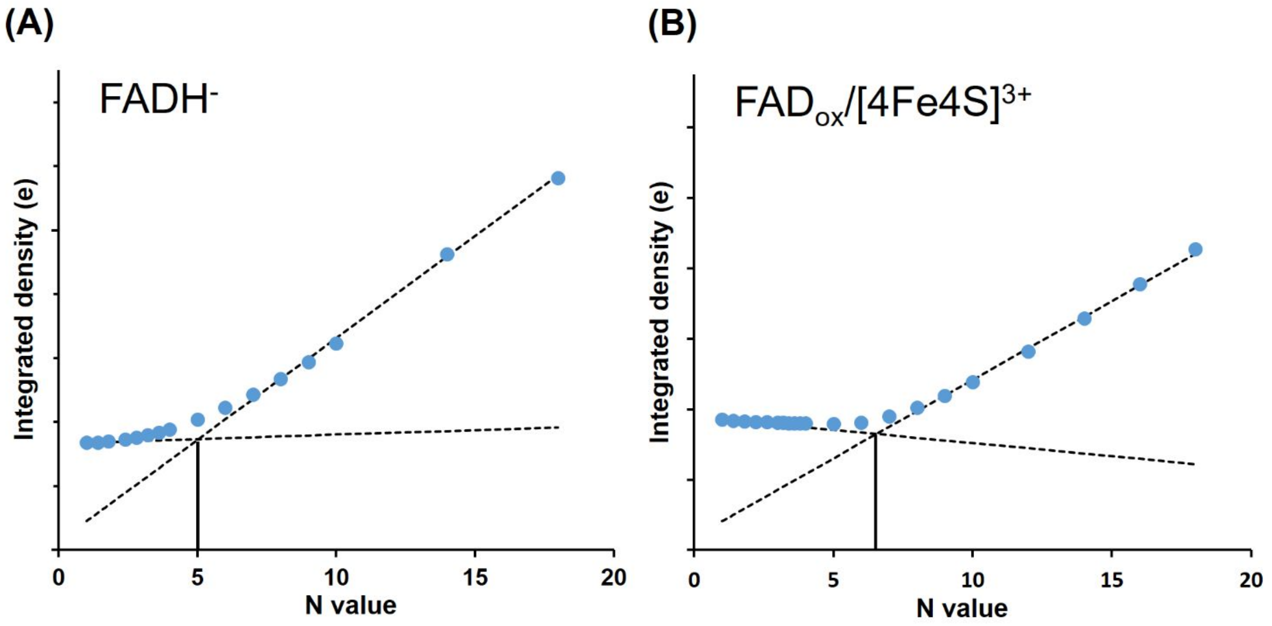

Figure S2. Estimation of redox state occupancy. Integrated residual negative densities plotted with extrapolation factor at (A) $FADH^-$ and (B) FAD_{ox}/$[4Fe4S]^{3+}$. The intersection between dashed linear trend lines was the N value used for the occupancy calculation and structure determination.

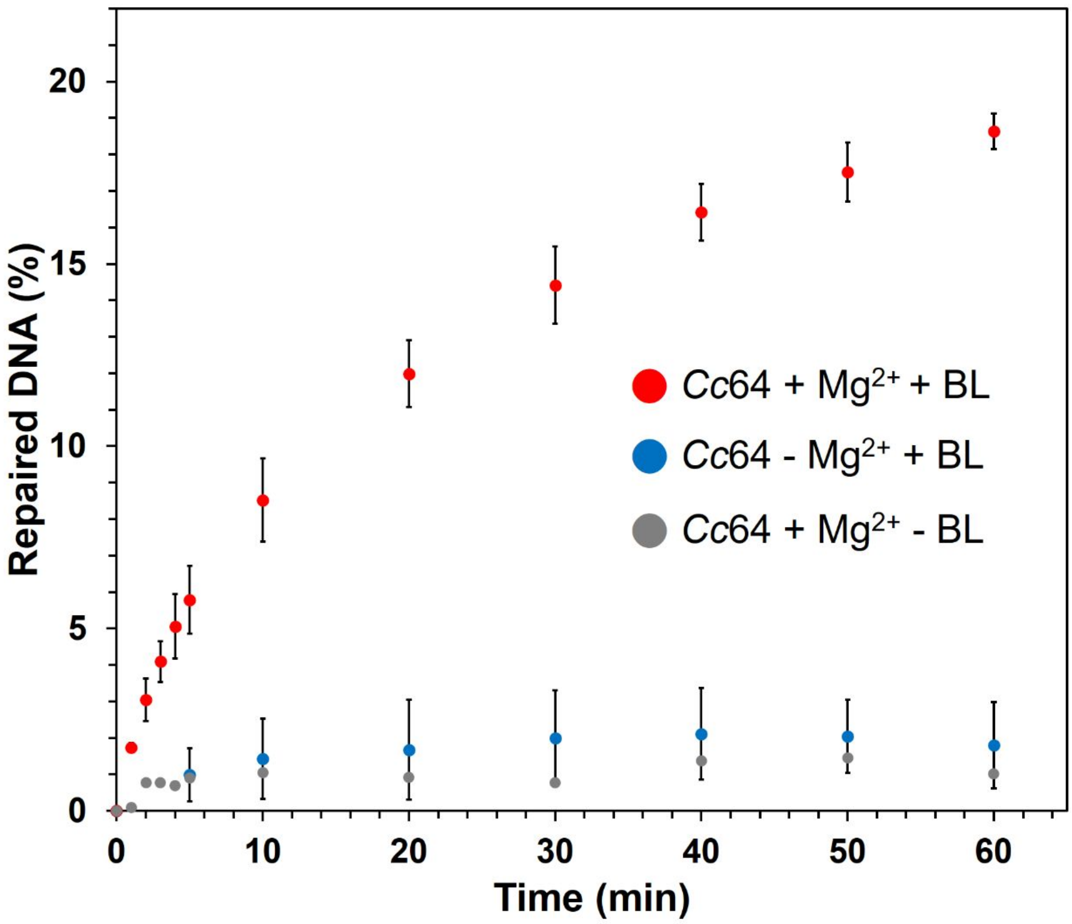

Figure S3. The *in vitro* DNA-repair assay was investigated to evaluate the efficacy of *Cc*(6-4)PL in repairing (6-4)PP lesions, and the impact of Mg^{2+} ions on the overall DNA repair process. The percentage of repaired (6-4)PP in ssDNA is provided for *in vitro* DNA repair without Mg^{2+} (blue dots) and with the addition of $MgCl_2$ (red dots). The negative control was conducted without light illumination (grey dots). Several time points were measured in a range from 0 min to 60 min illumination time. The data shown here indicate the mean value ± s.d. of three technical replicates.

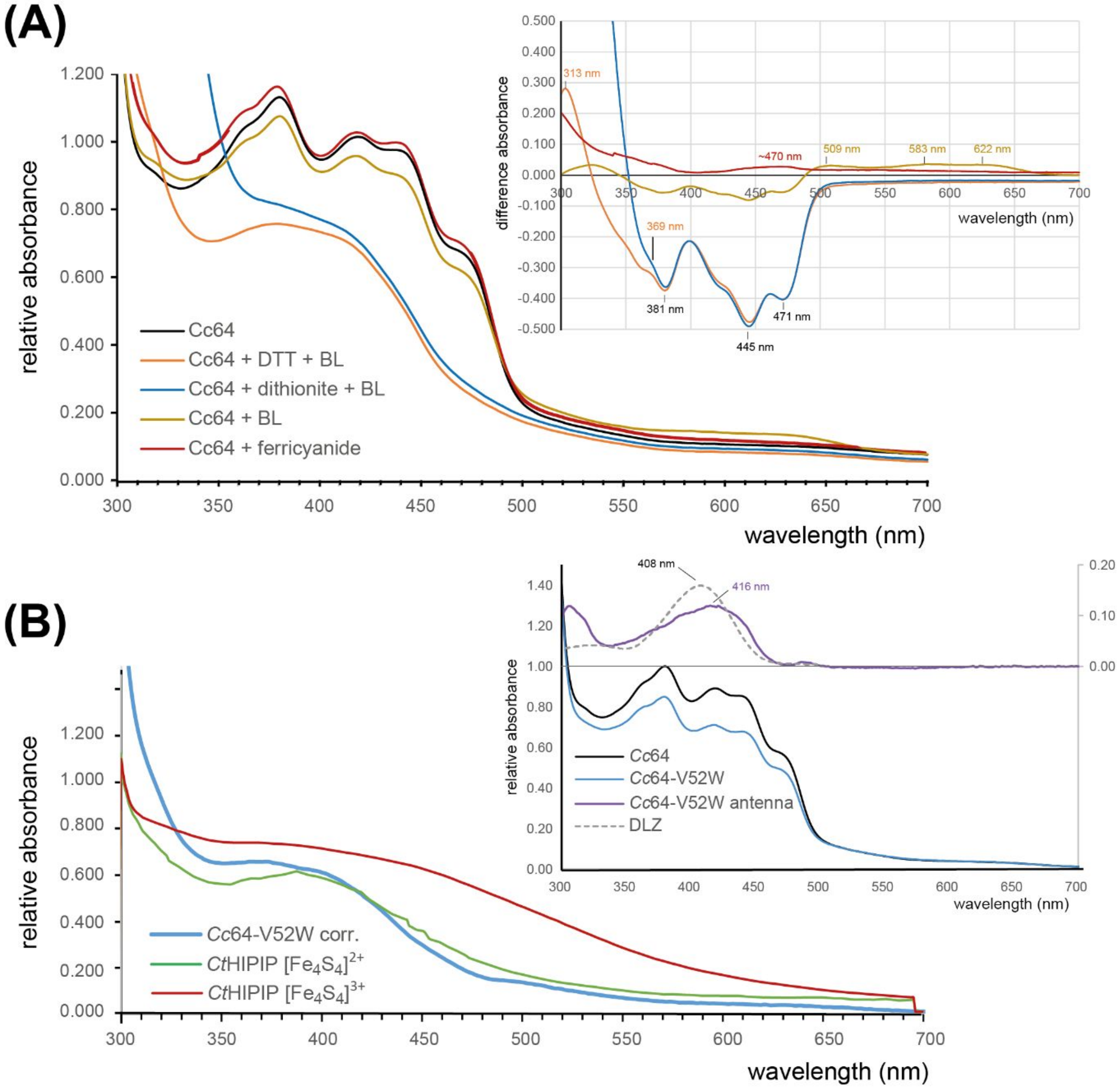

Figure S4. UV-Vis absorption spectroscopy of *Cc*(6-4)PL in its different redox states. (A) UV-Vis spectra of *Cc*(6-4)PL in all characterized redox states. *In vitro* photoreduction of *Cc*(6-4)PL from the FAD_{ox} state (black) was done in SEC buffer at 10°C resulting with blue light alone or with 50 mM DTT either the FADH• (gold) or the FADH– (orange) state; dithionite proved similarly efficient (blue). Oxidation of the iron-sulfur cluster to the $[4Fe\text{-}4S]^{3+}$ state (red) used 2 mM potassium ferricyanide for 3 minutes. The inlay shows the corresponding difference spectra relative to the FAD_{ox} state; difference peaks are labeled in colors corresponding to the respective redox states, e.g. black for FAD_{ox}. The spectral change at 470 nm upon oxidation to the $[4Fe\text{-}4S]^{3+}$ state corresponds to 6.7% of the change found upon the $FAD_{ox} \rightarrow FADH^{-}$ transition. (B) The V52W mutant lacks the DLZ antenna (inlay), thus simplifying the spectral analysis of the *Cc*(6-4) PL. Subtraction of the FAD_{ox} spectrum from the inlay of (A) gives a spectrum corresponding mainly to the iron-sulfur cluster of the V52W mutant. For comparison, the UV-Vis spectra of the *Chlorobium tepidum* HIPIP, *Ct*HIPIP, are shown for the $[4Fe\text{-}4S]^{2+}$ and $[4Fe\text{-}4S]^{3+}$ states as reported by Ohno et al.[5]. Notably, the $[4Fe\text{-}4S]^{2+}$ cluster contributes about 60% of the Cc(6-4) PL's light absorption at 390-400 nm.

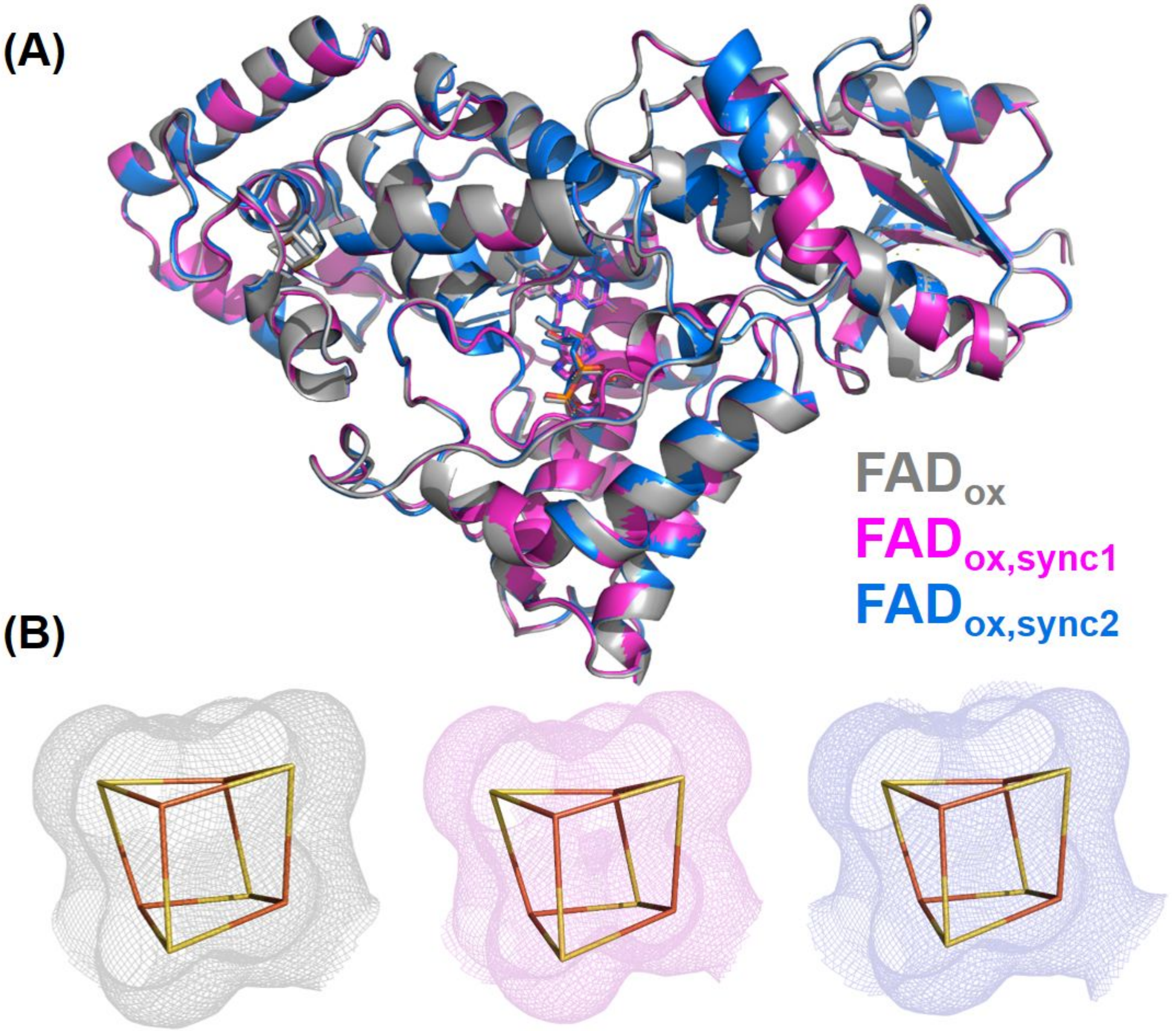

Figure S5. The overall oxidized *Cc*(6-4)PL structures. (A) The *Cc*(6-4)PL structures in FAD$_{ox}$, FAD$_{ox,sync1}$ and FAD$_{ox,sync2}$ are almost identical with r.m.s.d. values of 0.217 and 0.212 Å for 496 Cα positions, respectively. The overall structures are shown as cartoon models with FAD$_{ox}$, FAD$_{ox,sync1}$ and FAD$_{ox,sync2}$ in grey, magenta and blue, respectively. (B) SigmaA-weighted $2mF_{obs}$–DF_{calc} electron density map of oxidized state (FAD$_{ox}$) was contoured at 1.0 σ level. An intact electron density surrounding [4Fe-4S] in three structures and coloring as in panel A.

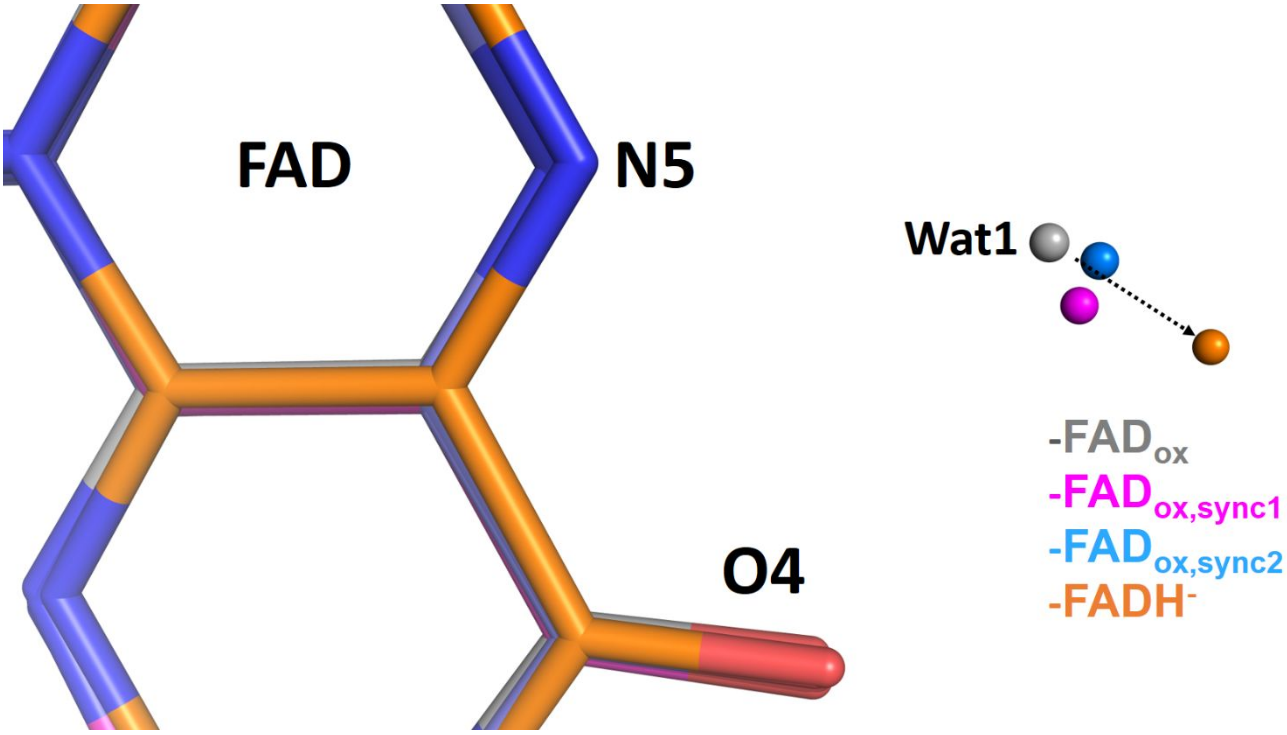

Figure S6. The water1 movement on three oxidized states and a reduced state. The FAD is shown as stick model and the water molecules are shown in the spheres model with FAD_{ox}, $FAD_{ox,sync1}$, $FAD_{ox,sync2}$ and $FADH^-$ in grey, magenta, blue and orange, respectively. The dashed arrow indicates movement between FAD_{ox} and $FADH^-$. The Wat1 of $FAD_{ox,sync1}$ and $FAD_{ox,sync2}$ are slightly shifted due to partial photoreduction by synchrotron X-ray irradiation. A strong movement in $FADH^-$ upon light illumination.

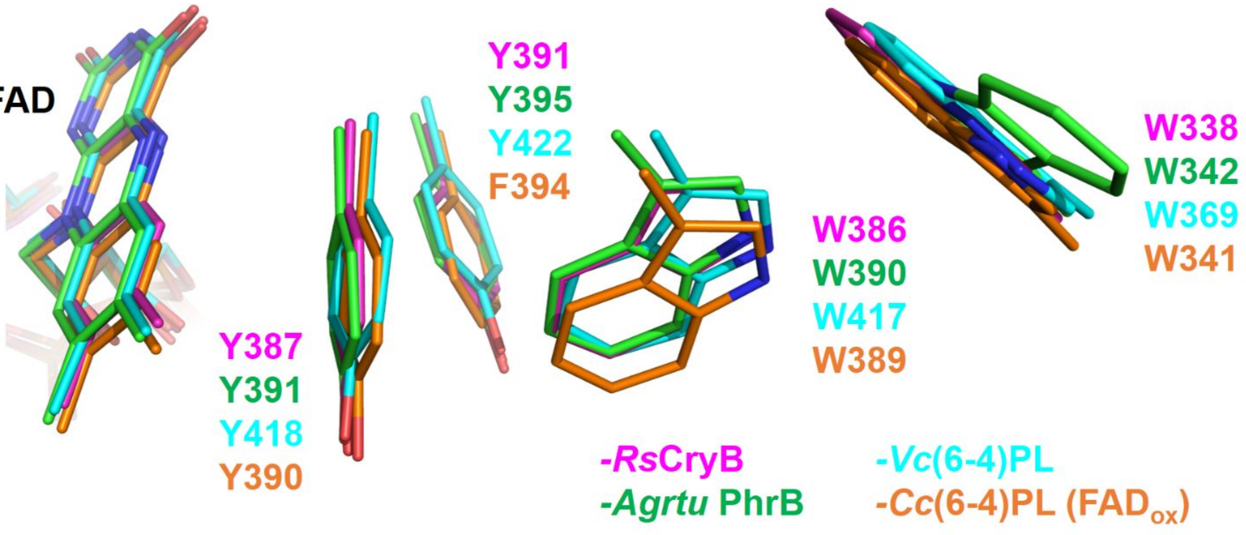

Figure S7. Structural superimposition of electron transfer chain in FeS-BCP members. The relative residues were shown by stick models. The *Cc*(6-4)PL was colored in orange, *Vc*(6-4)PL (PDB entry: 8A1H, cyan), *Agrtu*PhrB (PDB entry: 4DJA, green) and *Rs*CryB (PDB entry: 3ZXS, magenta). The proximal tyrosine (Y422) in *Vc*(6-4)PL is substituted by phenylalanine (F394) in *Cc*(6-4)PL.

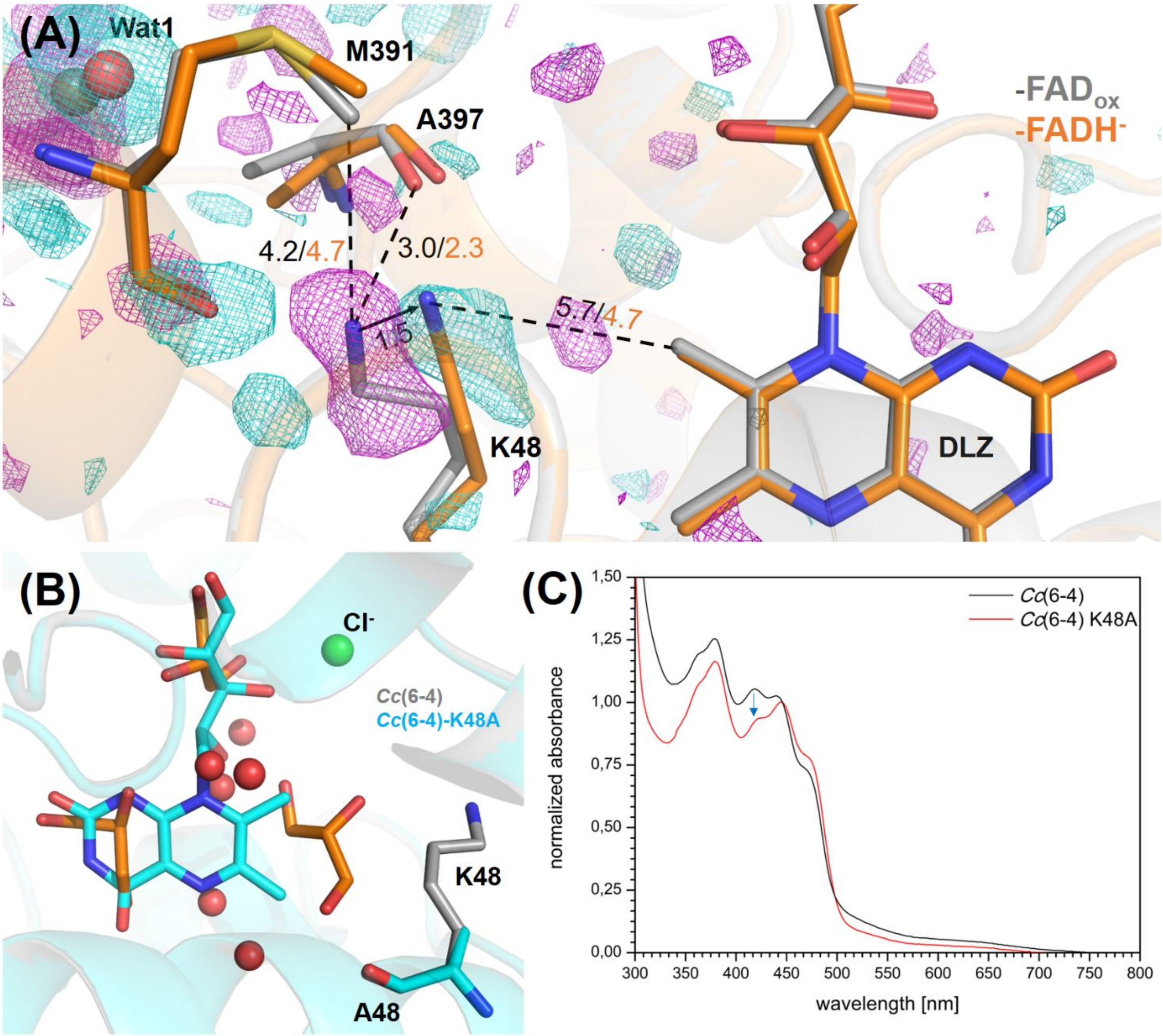

Figure S8. Structural comparison between different FAD_{ox} and $FADH^-$ around the DLZ binding pocket. (A) The reduced $FADH^-$ state of *Cc*(6-4)PL (orange) is shown in comparison to the FAD_{ox} state (grey). To highlight the experimental significance of the observed differences, 3.5 σ contoured DED maps are shown in cyan for positive peaks and magenta for negative ones. The sidechain of K48 moves towards DLZ in $FADH^-$. (B) DLZ binding pocket in *Cc*(6-4)PL-K48A mutant. The K48A mutant affected binding of the DLZ antenna, leading to its partial occupancy. The model was refined at 50% occupancy of DLZ (cyan), accompanied by an alternative DLZ-free state consisting of 3 glycerol molecules (orange), 6 waters (red sphere) and a chloride ion (green sphere). For *Cc*(6-4)PL-K48A, final statistics of data collection, processing, and refinement are summarized in Table S5. (C) Absorption spectra of *Cc*(6-4)PL WT (black) and K48A mutant (red) after size exclusion chromatography. To estimate the bound antenna amounts, absorption maxima at 418 nm were compared. The antenna of the K48A mutant is lost by more than 20% relative to WT (arrow at 418 nm). Further loss of the antenna may be due to the crystallization and cryoprotection conditions.

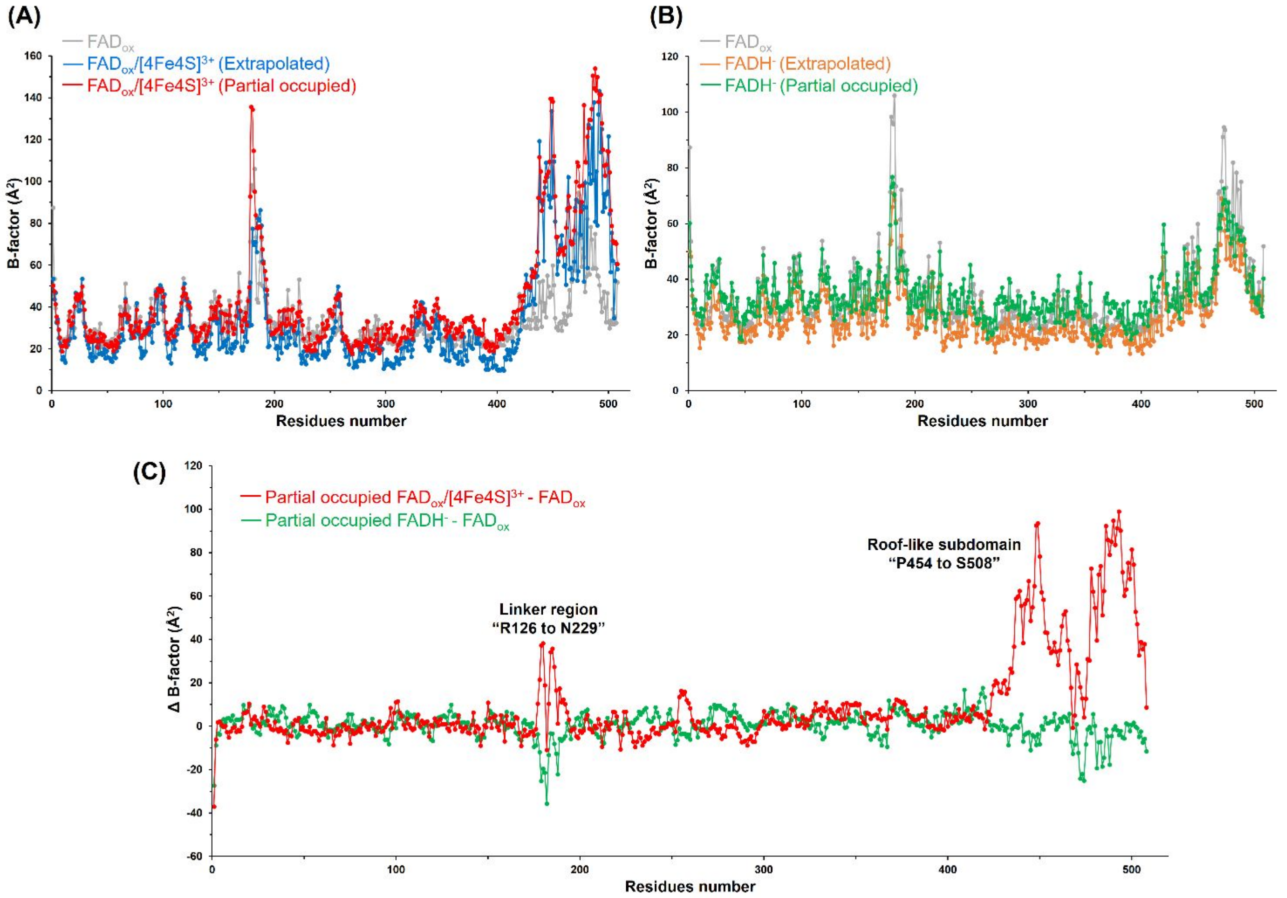

Figure S9. B-factor distributions of *Cc*(6-4)PL residues in its different redox states as obtained from SFX data. (A) B-factor distributions are compared between the $FAD_{ox}/[4Fe4S]^{2+}$ state as obtained from FAD_{ox} SFX data (grey), the superoxidized $FAD_{ox}/[4Fe4S]^{3+}$ state as obtained either from extrapolated data (blue) or from non-extrapolated data (red). The latter assumed an occupancy of 34%. (B) B-factor distribution of the FAD_{ox} state (grey), the $FADH^{-}$ state from extrapolated (orange) and non-extrapolated data (40%, green). (C) Difference B-factor plots were calculated by subtracting FAD_{ox} B-factors from non-extrapolated $FAD_{ox}/[4Fe4S]^{3+}$ (red) and $FADH^{-}$ (green) states. Notably, the roof-like subdomain exhibits significant destabilization upon oxidation of the [4Fe4S] cluster, indicating increased structural disorder.

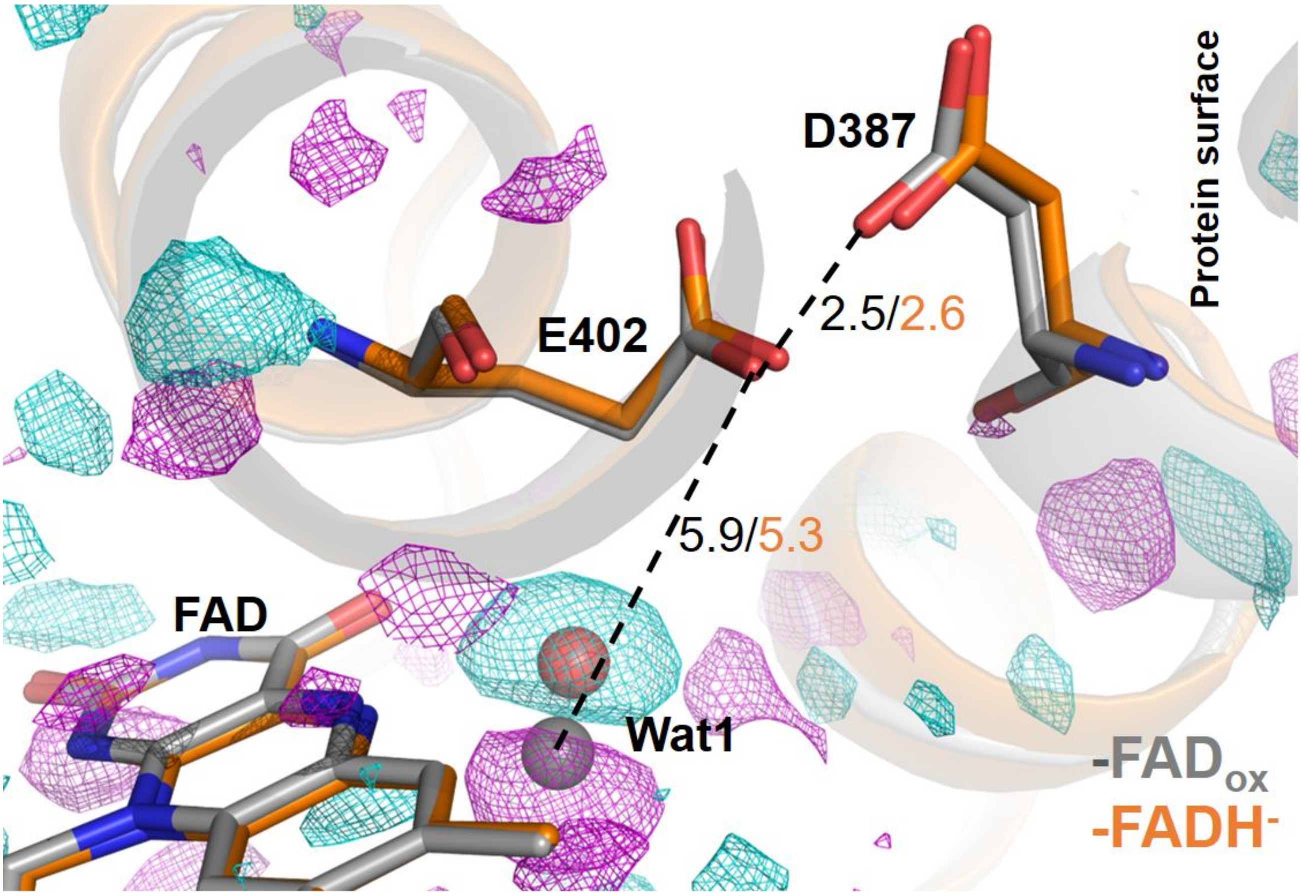

Figure S10. Structural comparison between FAD redox states along the putative protonation pathway. The reduced $FADH^-$ state of *Cc*(6-4)PL (orange) is shown in comparison to the FAD_{ox} state (grey). To highlight the experimental significance of the observed differences, 3.5 σ contoured DED maps are shown in cyan for positive peaks and magenta for negative ones. Apart from the movement of Wat1, only minor changes are apparent for E402 and D387.

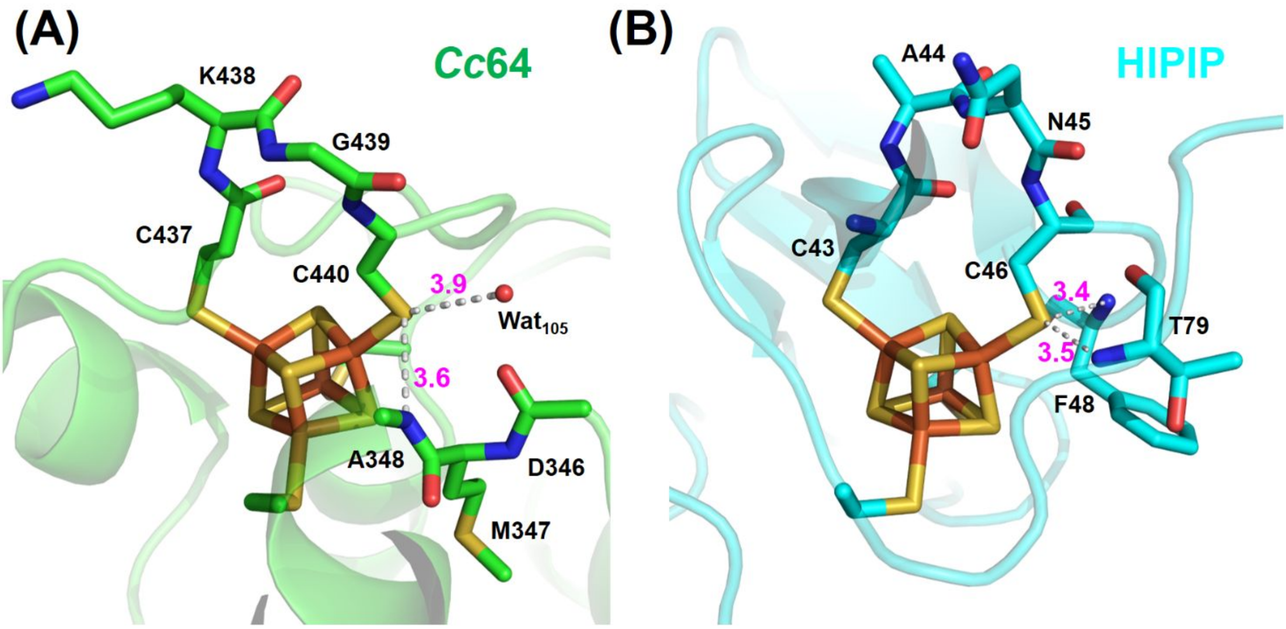

Figure S11. Comparison of the [4Fe-4S] clusters in *Cc*(6-4)PL and HIPIP from *Chromatium tepidum*. (A) The structural similarity between the C437-C440 loop in *Cc*(6-4)PL and (B) the C43-C46 loop in HIPIP (PDB entry: 5WQR)[6] is depicted using green and cyan stick models, respectively. Notably, only the Sγ atom of the second cysteine of the CxxC motif (sticks) makes hydrogen bonds to donor groups. Despite the different structural context, the CxxC motifs adopt almost the same conformation. Hydrogen bond distances (dashed line, grey) are indicated in angstroms (Å) and labelled in magenta.

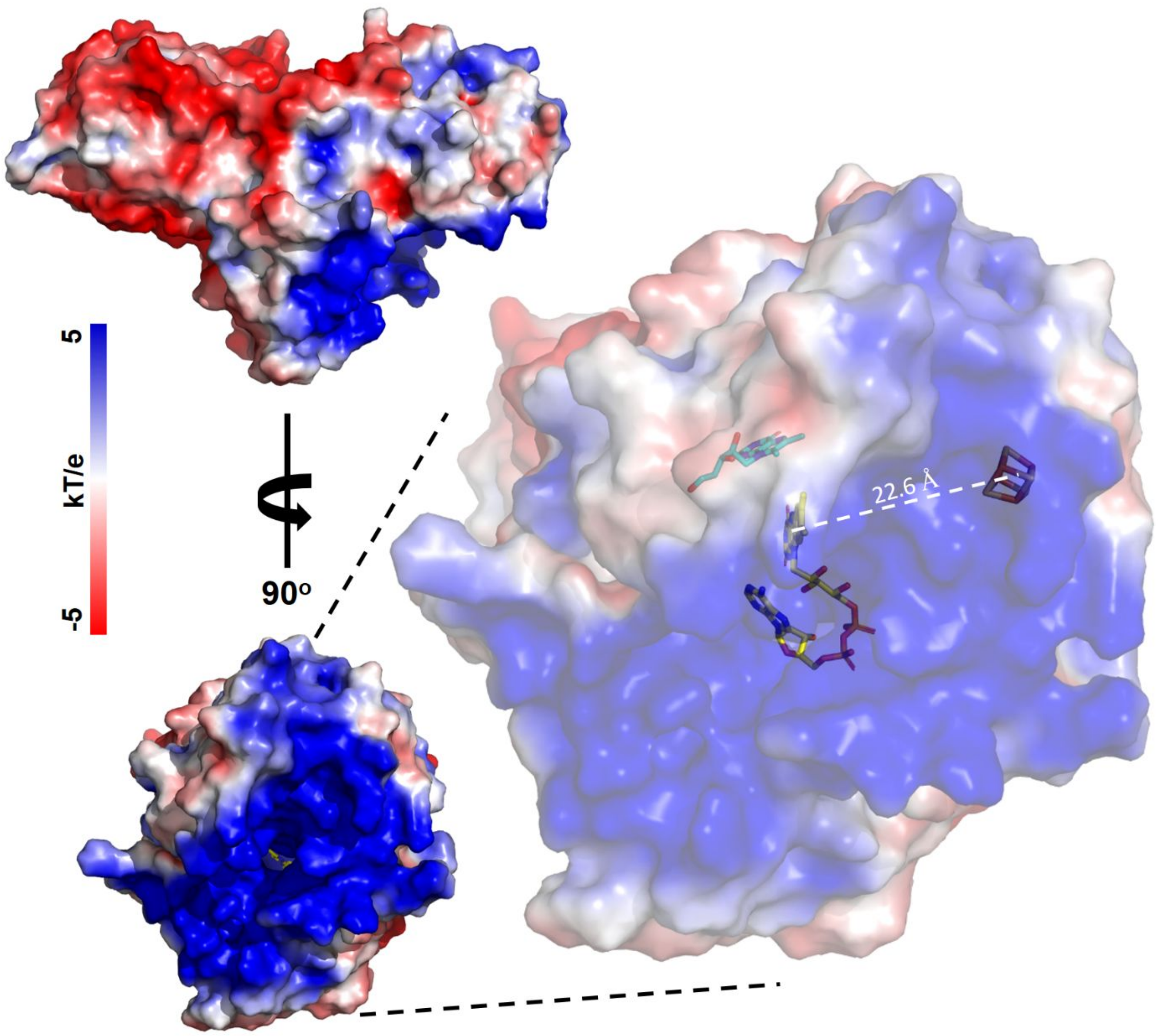

Figure S12. Electrostatic surface potential of the *Cc*(6-4) photolyase. The electrostatics was calculated by APBS[7] and mapped onto the molecular surface as shown with 50% transparency (blue: positive; white: neutral; red: negative). The DNA-binding site of *Cc*(6-4)PL corresponds to the positively charged groove around its active site. This binding site positions the DNA (6-4) lesions into the active site for direct interaction with the adenine moiety of the catalytic FAD cofactor (yellow). The [4Fe-4S] cluster is located close to the enzyme's surface interacting with double-stranded DNA. The centroid-centroid distance between the [4Fe-4S] cluster and the FAD isoalloxazine moiety is shown as dashed line.

Supporting Tables

Table S1. Synchrotron and SFX data statistics for steady-state structures. Numbers in parenthesis describe the highest resolution shell.

***Cc*(6-4)PL structures**	**$FAD_{ox,sync1}$**	**$FAD_{ox,sync2}$**	**FAD_{ox}**	**$FADH^{-}$**	**$FAD_{ox}/[4Fe\text{-}4S]^{3+}$**
PDB code	9HNK	9HNL	9HNM	9HNN	9HNO
Beamline	SLS-X06SA	TPS-05A	SACLA-BL2	SACLA-BL2	SACLA-BL2
Wavelength (Å)	1.0	1.0	1.24	1.24	1.24
Temperature (K)	100	293	293	293	293
Space group	$P2_12_12_1$				
Indexed crystals	1	1	74470	51116	59143
Unit cell (*a*, *b*, *c*)*	49.3,102.7,103.9	49.3,102.7,103.5	49.8,103.4,104.8	49.8,103.4,104.8	49.8,103.4,104.8
Processing statistics					
Resolution range (Å)	49.27-1.59 (1.67-1.59)	46.23-2.20 (2.32-2.20)	32.74-1.71 (1.72-1.71)	29.60-1.78# (1.80-1.78)	32.74-1.84 (1.86-1.84)
Completeness (%)	99.53 (99.94)	99.59 (99.96)	99.94 (100.00)	99.93 (100.00)	99.85 (100.00)
Observations	737710	110541	70527506	44484637	52103617
Unique reflections	72217 (7106)	27347 (2690)	59370 (5836)	52730 (5187)	47810 (2436)
Multiplicity	10.2 (10.3)	4.0 (4.1)	1188 (112)	843 (162)	1090 (188)
CC1/2	0.997 (0.52)	0.996 (0.524)	0.998 (0.5)	0.997 (0.544)	0.998 (0.524)
I/σ	8.3 (1.41)	7.5 (1.89)	11.38 (1.49)	9.46 (1.39)	10.55 (1.49)
R_{merge}	0.114 (1.749)	0.220 (1.065)	0.069 (1.134).	0.087 (1.132)	0.080 (1.095)
State occupancy (%)	100	100	100	40	33
Refinement statistics					
Resolution range (Å)	46.36-1.59 (1.65-1.59)	44.48-2.20 (2.28-2.20)	24.23-1.71 (1.77-1.71)	24.9-1.78# (1.84-1.78)	24.9-2.30# (2.38-2.30)
Unique reflections	71601 (7105)	27334 (2689)	59278 (5836)	52642 (5187)	24732 (2436)
R-work	0.1808 (0.2786)	0.1933 (0.2594)	0.1589 (0.2518)	0.1839 (0.3846)	0.2171 (0.3509)
R-free	0.2019 (0.2926)	0.2377 (0.2812)	0.1877 (0.2562)	0.2123 (0.4170)	0.2648 (0.3641)
Ramachandran outliers (%)	0	0	0	0.4	0.99
Number of non-H atoms	4731	4504	4527	4480	4453
RMS (bonds, Å)	0.015	0.002	0.017	0.006	0.002
RMS (angles, deg)	1.24	0.53	1.45	0.96	0.49
Average *B* factor	28.17	29.31	34.67	28.06	33.48

*α=β=γ=90°

#Resolution values given here correspond to the extrapolated structure factor dataset.

Table S2. Map correlation values between observed and calculated DED maps on the residues.

Position	dFo vs. dFc ($FADH^-$)	dFo vs. dFc (FAD_{ox}/[4Fe-4S]$^{3+}$)
[4Fe4S]	0.8074	0.8377
C349	0.7418	0.7405
C437	0.5658	0.7376
C440	0.8059	0.8068
C453	0.5284	0.7982
W341	0.5462	0.6305
W389	0.7223	0.5145
Y390	0.8313	0.6208
F394	0.8017	0.6506

Table S3. The distances of atoms were shown in angstrom within iron sulfur cluster in damage-free structures.

Atom - Atom	FAD_{ox}	$FADH^-$	FAD_{ox}/ $[4Fe\text{-}4S]^{3+}$
Fe1 – C437	2.296	2.255	2.274
Fe2 – C453	2.305	2.280	2.262
Fe3 – C349	2.270	2.302	2.286
Fe4 – C440	2.273	2.230	2.998
Average ± std	2.286 ± 0.017	2.267 ± 0.031	2.274 ± 0.012#
Fe1 – Fe2	2.727	2.761	2.740
Fe1 – Fe3	2.734	2.707	2.739
Fe1 – Fe4	2.772	2.756	2.737
Fe2 – Fe3	2.786	2.711	2.738
Fe2 – Fe4	2.726	2.724	2.737
Fe3 – Fe4	2.752	2.783	2.733
Average ± std	2.749 ± 0.023	2.740 ± 0.028	2.737 ± 0.002
Fe1 – S2	2.277	2.248	2.280
Fe1 – S3	2.293	2.308	2.277
Fe1 – S3	2.257	2.29	2.284
Fe2 – S1	2.289	2.257	2.285
Fe2 – S3	2.252	2.272	2.272
Fe2 – S4	2.296	2.313	2.289
Fe3 – S1	2.314	2.302	2.283
Fe3 – S2	2.243	2.282	2.278
Fe3 – S4	2.264	2.253	2.286
Fe4 – S1	2.254	2.258	2.276
Fe4 – S2	2.298	2.314	2.277
Fe4 – S3	2.271	2.257	2.285
Average ± std	2.276 ± 0.021	2.279 ± 0.024	2.281 ± 0.005
S1 –S2	3.585	3.576	3.595
S1 –S3	3.575	3.561	3.595
S1 –S4	3.582	3.555	3.607
S2 – S3	3.580	3.599	3.598
S2 – S4	3.553	3.570	3.598
S3 – S4	3.602	3.578	3.594
Average ± std	3.580 ± 0.015	3.593 ± 0.021	3.598 ± 0.004

#The Fe4 – C440 distance was excluded for calculation of average number and standard deviation.

Table S4. State-dependent structural distortions of the FAD's isoalloxazine moiety.

Protein	Redox state	PDB	ρC (°)	ρN (°)	#Butterfly bending (°)
Cc(6-4)PL	$FAD_{ox,sync1}$	9HNK	0.5	0.6	0.1
Cc(6-4)PL	$FAD_{ox,sync2}$	9HNL	0.4	0.5	0.1
Cc(6-4)PL	FAD_{ox}	9HNM	0.2	0.1	1.1
Cc(6-4)PL	$FADH^-$	9HNN	5.1	6.8	5.2
Cc(6-4)PL	FAD_{ox}/$[4Fe4S]^{3+}$	9HNO	0.04	0.02	0.04
*An*CPDI	※$FADH^-$	1TEZ	8.8	9.5	9
*Mm*CPDII	$FADH^-$	7VJ7	14.3	14.5	16.3
*Mm*CPDII	FAD_{ox}→$FAD^{-\bullet}$ (125 µs*)	7VJ2	20.3	18.8	18.4
Dm(6-4)PL	FAD_{ox}→$FAD^{-\bullet}$ (100 µs*)	8C69	0.8	1.4	2.4

*The structure was obtained from time-resolved SFX datasets[8-9].
※The structure was obtained from synchrotron data[10]. In this structure, radiation damage apparently caused repair of the bound CPD lesion and reduction of the FAD chromophore due to photoelectrons.
#The butterfly-like bending angle of the isoalloxazine ring is calculated by measuring the angle between the two normal vectors over the planes spanned by the benzo (C5x-C6-C7-C8-C9-C9A) and pyrimidino (N1-C2-N3-C4-C4X-C10) rings of the isoalloxazine moiety. Matrix and linear algebra calculations were done within an Excel® spreadsheet (Microsoft).

Table S5. Synchrotron data statistics for *Cc*64PL-K48A structure. Numbers in parenthesis describe the highest resolution shell.

**Structures**	***Cc*(6-4)PL-K48A$_{dark}$**
PDB code	9Q8F
Beamline	ESRF, ID23-2
Wavelength (Å)	0.87
Temperature (K)	100
Space group	$P2_12_12_1$
Indexed crystals	1
Unit cell (*a*, *b*, *c*)*	48.98, 102.1, 102.94
Processing statistics	
Resolution range (Å)	45.96 - 1.50 (1.53 - 1.50)
Completeness (%)	98.69 (94.29)
Observations	984030 (88700)
Unique reflections	82472 (7750)
Multiplicity	11.9 (11.4)
CC1/2	0.993 (0.905)
I/σ	21.94 (4.95)
R_{merge}	0.0907 (0.6294)
Refinement statistics	
DLZ occupancy (%)	50
Resolution range (Å)	45.74 - 1.50 (1.55 - 1.50)
Unique reflections	82225 (7746)
R-work	0.1381 (0.1980)
R-free	0.1646 (0.2136)
Ramachandran outliers (%)	0
Number of non-H atoms	5001
RMS (bonds, Å)	0.009
RMS (angles, deg)	1.00
Average *B* factor	21.29

*α=β=γ=90°
#Resolution values given here correspond to the extrapolated structure factor dataset.

References

1. Mäntele, W.; Deniz, E., UV–VIS absorption spectroscopy: Lambert-Beer reloaded. *Spectrochimica Acta Part A: Molecular and Biomolecular Spectroscopy* **2017,** *173*, 965-968.

2. Terai, Y.; Sato, R.; Yumiba, T.; Harada, R.; Shimizu, K.; Toga, T.; Ishikawa-Fujiwara, T.; Todo, T.; Iwai, S.; Shigeta, Y.; Yamamoto, J., Coulomb and CH–π interactions in (6–4) photolyase–DNA complex dominate DNA binding and repair abilities. *Nucleic acids research* **2018,** *46* (13), 6761-6772.

3. Brych, A.; Mascarenhas, J.; Jaeger, E.; Charkiewicz, E.; Pokorny, R.; Bölker, M.; Doehlemann, G.; Batschauer, A., White collar 1-induced photolyase expression contributes to UV-tolerance of Ustilago maydis. *Microbiologyopen* **2016,** 5 (2), 224-243.

4. Vallejos, A.; Katona, G.; Neutze, R., Appraising protein conformational changes by resampling time-resolved serial x-ray crystallography data. *Structural dynamics (Melville, N.Y.)* **2024,** *11* (4), 044302.

5. Ohno, H.; Takeda, K.; Niwa, S.; Tsujinaka, T.; Hanazono, Y.; Hirano, Y.; Miki, K., Crystallographic characterization of the high-potential iron-sulfur protein in the oxidized state at 0.8 Å resolution. *PloS one* **2017,** *12* (5), e0178183.

6. Grunwald, L.; Abbott, D. F.; Mougel, V., Gauging Iron–Sulfur Cubane Reactivity from Covalency: Trends with Oxidation State. *JACS Au* **2024,** *4* (4), 1315-1322.

7. Jurrus, E.; Engel, D.; Star, K.; Monson, K.; Brandi, J.; Felberg, L. E.; Brookes, D. H.; Wilson, L.; Chen, J.; Liles, K.; Chun, M.; Li, P.; Gohara, D. W.; Dolinsky, T.; Konecny, R.; Koes, D. R.; Nielsen, J. E.; Head-Gordon, T.; Geng, W.; Krasny, R.; Wei, G. W.; Holst, M. J.; McCammon, J. A.; Baker, N. A., Improvements to the APBS biomolecular solvation software suite. *Protein science : a publication of the Protein Society* **2018,** *27* (1), 112-128.

8. Maestre-Reyna, M.; Yang, C.-H.; Nango, E.; Huang, W.-C.; Ngurah Putu, E. P. G.; Wu, W.-J.; Wang, P.-H.; Franz-Badur, S.; Saft, M.; Emmerich, H.-J.; Wu, H.-Y.; Lee, C.-C.; Huang, K.-F.; Chang, Y.-K.; Liao, J.-H.; Weng, J.-H.; Gad, W.; Chang, C.-W.; Pang, A. H.; Sugahara, M.; Owada, S.; Hosokawa, Y.; Joti, Y.; Yamashita, A.; Tanaka, R.; Tanaka, T.; Luo, F.; Tono, K.; Hsu, K.-C.; Kiontke, S.; Schapiro, I.; Spadaccini, R.; Royant, A.; Yamamoto, J.; Iwata, S.; Essen, L.-O.; Bessho, Y.; Tsai, M.-D., Serial crystallography captures dynamic control of sequential electron and proton transfer events in a flavoenzyme. *Nature Chemistry* **2022,** *14* (6), 677-685.
